# Optimising the sensitivity of optically-pumped magnetometer magnetoencephalography to gamma band electrophysiological activity

**DOI:** 10.1162/imag_a_00112

**Published:** 2024-03-19

**Authors:** Ryan M. Hill, Holly Schofield, Elena Boto, Lukas Rier, James Osborne, Cody Doyle, Frank Worcester, Tyler Hayward, Niall Holmes, Richard Bowtell, Vishal Shah, Matthew J. Brookes

**Affiliations:** Sir Peter Mansfield Imaging Centre, School of Physics and Astronomy, University of Nottingham, University Park, Nottingham, United Kingdom; Cerca Magnetics Limited, Nottingham, United Kingdom; QuSpin, Inc., Louisville, Colorado, United States

**Keywords:** optically-pumped-magnetometer, OPM, magnetoencephalography, MEG, sensitivity, beamformer

## Abstract

The measurement of electrophysiology is of critical importance to our understanding of brain function. However, current non-invasive measurements—electroencephalography (EEG) and magnetoencephalography (MEG)—have limited sensitivity, particularly compared to invasive recordings. Optically-Pumped Magnetometers (OPMs) are a new type of magnetic field sensor which ostensibly promise MEG systems with higher sensitivity; however, the noise floor of current OPMs remains high compared to cryogenic instrumentation and this limits the achievable signal-to-noise ratio of OPM-MEG recordings. Here, we investigate how sensor array design affects sensitivity, and whether judicious sensor placement could compensate for the higher noise floor. Through theoretical analyses, simulations, and experiments, we use a beamformer framework to show that increasing the total signal measured by an OPM array—either by increasing the number of sensors and channels, or by optimising the placement of those sensors—affords a linearly proportional increase in signal-to-noise ratio (SNR) following beamformer reconstruction. Our experimental measurements confirm this finding, showing that by changing sensor locations in a 90-channel array, we could increase the SNR of visual gamma oscillations from 4.8 to 10.5. Using a 180-channel optimised OPM-array, we capture broadband gamma oscillations induced by a naturalistic visual paradigm, with an SNR of 3; a value that compares favourably to similar measures made using conventional MEG. Our findings show how an OPM-MEG array can be optimised to measure brain electrophysiology with the highest possible sensitivity. This is important for the design of future OPM-based instrumentation.

## 1 Introduction

The measurement of electrophysiological activity in the human brain offers unique insights into brain function and its breakdown in disease. In the healthy brain, electrophysiological signals are dominated by neural “oscillations” which comprise repetitive fluctuations in electrical potential, measurable across assemblies of neurons (Pfurtscheller & Lopes da Silva, 1999). Such oscillations have proven to be integral to our understanding of brain function; for example, significant evidence suggests that they help mediate both short- and long-range connectivity between functionally related areas (Sadaghiani et al., 2022). Abnormal electrical activity provides a biomarker of neurological conditions, with examples including “spike and wave” activity in epilepsy (Rampp et al., 2019), or cortical slowing (i.e., the abnormal dominance of low-frequency oscillations) in dementia (Engels et al., 2017). Consequently, the characterisation of electrophysiological phenomena—in health and disease—is of high importance. The highest accuracy electrophysiological measurement involves electrodes placed directly on or beneath the brain surface, which measure fluctuating electrical potential—a technique called intracranial electroencephalography (iEEG) (Almeida et al., 2005). iEEG allows assessment of electrical activity with the best possible sensitivity, excellent (millisecond) temporal resolution, and spatial resolution at the millimetre scale. However, iEEG is highly invasive—requiring major surgery—and this limits its use to patients (e.g., with intractable epilepsy) or experimental animals. It also cannot achieve wide coverage (you cannot place electrodes everywhere) and measurements are very sensitive to electrode placement (due to the focality of the electrodes’ lead fields). If a means could be found to achieve a sensitivity approaching that of iEEG, but using non-invasive sensors, this could be extremely valuable for basic neuroscience, clinical research, and management of patients with neurological and psychiatric conditions.

The most common non-invasive technique to map electrophysiological activity is EEG, where electrodes are placed in conductive contact with the scalp (Berger, 1929). EEG is ubiquitous in clinical settings, providing measurements of electrophysiology with high temporal resolution. However, sensitivity is limited (compared to iEEG) for two reasons: first, the skull has a high electrical resistance which dramatically reduces the amplitude of signals reaching the scalp; second, EEG is sensitive to physiological signals of no interest, for example, those generated by muscles which propagate through the skin to EEG electrodes via conduction. These artefacts have impact across the whole spectrum but are particularly problematic at frequencies above ~20 Hz where brain signals tend to be lower amplitude (Whitham et al., 2007,^2008^). These problems are alleviated, in part, by magnetoencephalography (MEG) which measures the magnetic fields generated by the same neural current flows that give rise to iEEG and EEG signals (Baillet, 2017;Cohen, 1968). Because the magnetic permeability of the head is approximately uniform (and similar to that of free space), MEG signals are not attenuated by the skull in the same way as EEG. MEG is also less sensitive to muscle artefacts than EEG (Boto et al., 2019;Claus et al., 2012;Muthukumaraswamy, 2013), giving higher sensitivity at high frequency. However, most MEG systems rely on superconducting sensors which require very low temperatures, and so must be housed at a minimum distance of ~2 cm from the head to accommodate a thermally insulating vacuum. Because the MEG signal amplitude falls with the square of distance, this large source to sensor separation, alongside an inherent sensor noise floor of ~2-5 fT/√Hz, reduces the achievable sensitivity. In sum, both EEG and MEG have fundamental physical limitations on the achievable sensitivity to electrophysiological effects, with neither able to compete with iEEG.

In recent years, optically-pumped magnetometers (OPMs) have been developed as an alternative to superconducting sensors for MEG (Brookes et al., 2022;Schofield et al., 2023;Xia et al., 2006). OPMs are small and lightweight magnetic field sensors, which do not require cryogenic cooling and consequently can be positioned much closer to the scalp surface than cryogenic sensors. This improvement in proximity means that OPMs detect a magnetic field that is ~4-5 times larger in amplitude (for cortical sources) compared to conventional MEG (Boto et al., 2016;Iivanainen et al., 2017). Ostensibly, this may translate to a higher sensitivity, however the broadband sensitivity to noise of most practical OPMs remains higher than that of a superconducting sensor (for single-axis OPMs, the noise floor is ~7-12 fT/√Hz with a bandwidth of 130 Hz; for triaxial OPMs, noise floors are ~13-15 fT/√Hz) (Boto et al., 2022). This means that, while improved proximity has enabled OPM-MEG to experimentally demonstrate improved sensitivity compared to conventional-MEG (Boto et al., 2017;Feys et al., 2022), those improvements are (in part) masked by increased sensor noise.

At a superficial level, it therefore appears that the sensitivity of OPM-MEG also has intrinsic limitations. However, the above arguments offer only a simplistic picture since, in practice, the sensitivity of a MEG system is governed not only by sensor proximity and noise, but also by system design and dominant interference. Most MEG studies aim to combine signals measured across sensor array, using a weighted integral to estimate electrophysiological activity at some location in the brain—a process called linear source localisation. By combining signals from multiple sensors, the contribution of sensor noise can be supressed; the more sensors there are, the more effective that suppression will be (Vrba et al., 2004). Sensitivity is also strongly affected by the characteristics of the dominant sources of unwanted signal; this may be intrinsic sensor noise but could equally be magnetic fields generated in the local environment (e.g., by laboratory equipment), fields generated by other organs in the body (e.g., the heart), or fields from sources of no interest in the brain. In these latter cases, sensitivity becomes critically dependant on how well we can differentiate fields generated by sources internal and external to the head (in the case of environmental noise) (Taulu et al., 2005) or how well we can distinguish between brain locations. When taking into account these additional factors, OPM-MEG gains significant advantages: Careful OPM array design offers advantages in the rejection of external interference (Brookes et al., 2021;Tierney et al., 2022), and the flexibility to locate sensors anywhere on the head surface should enable optimisation of an array to diminish the effects of sensor noise and maximise sensitivity (Nugent et al., 2022).

In this paper, we characterise the way in which sensitivity is affected by sensor array design. We focus on high-frequency effects, because these are the most challenging to measure due to their low amplitude. Previous work on OPM sensor arrays have focussed on dipole fitting (Beltrachini et al., 2021) and minimum norm estimation (MNE) (Takeda et al., 2023) as mathematical frameworks for source localisation. Here we develop our theory within a beamformer framework (Robinson & Vrba, 1998)—beamforming is a verifiable source localisation strategy, which has become popular for the characterisation of neural oscillations (Hillebrand et al., 2005). In what follows, we first develop an analytical framework to characterise how array design impacts reconstruction error. We then undertake simulations to show the viability of this analytic model. In the second part of the paper, we describe experimental measurements, demonstrating how it is possible to optimise sensitivity to high-frequency (gamma-band) neural oscillatory processes in human visual cortex.

## 2 Theory

The generative model for MEG states that the magnetic fields,b(t), produced by a single dipolar current source of amplitude$q_{\alpha }\left(t\right)$, and measured at time*t*(using*M*MEG channels) is given by,

$$\text{b}\left(t\right)={\text{L}}_{\alpha }q_{\alpha }\left(t\right)+\text{e}\left(t\right)$$. [1]

Here,${\text{L}}_{\alpha }$is an M x 1 vector delineating the forward field for a source with location and orientation,α.e(t)reflects the measurement error—we assume this to be generated by sensor noise, which is uncorrelated across channels.

Using a beamformer, we aim to invertEquation 1by estimating source strength,$\hat{q_{\theta }}\left(t\right)$at some location and orientation,θ(which may or may not be the same as the true source location,α) based on the measured magnetic field. We assume that the estimate can be given as a weighted sum of the sensor measurements such that

$$\hat{q_{\theta }}\left(t\right)={\text{w}}_{\theta }^{T}\text{b}\left(t\right)$$. [2]

The weighting parameters,${\text{w}}_{\theta }$, tuned toθ, are derived based upon the minimisation of the output source variance, subject to a linear constraint that source power generated atθmust remain in the output signal. Mathematically, this problem can be cast as

$$\underset{{\text{w}}_{\theta }}{\min}\langle {\hat{q_{\theta }}}^{2}\rangle \mathrm{subject}\;\mathrm{to}\;{\text{w}}_{\theta }^{T}{\text{L}}_{\theta }=1$$, [3]

(where〈x〉denotes the expectation value ofx). A solution is

$${\text{w}}_{\theta }^{T}=\frac{{\text{L}}_{\theta }^{\text{T}}{\text{C}}^{-1}}{{\text{L}}_{\theta }^{\text{T}}{\text{C}}^{-1}{\text{L}}_{\theta }}$$, [4]

whereCrepresents the data covariance matrix (i.e.,$\text{C}=\langle \text{b}{\text{b}}^{\text{T}}\rangle$). If we assume that the beamformer estimate is made at the true source location such that${\text{L}}_{\theta }\approx {\text{L}}_{\alpha }$then, by subsitiutingequation 1intoequation 2, and noting again the linear constraint,${\text{w}}_{\theta }^{T}{\text{L}}_{\theta }=1$, we find that

$$\hat{q_{\alpha }}\left(t\right)={\text{w}}_{\alpha }^{T}{\text{L}}_{\alpha }q_{\alpha }\left(t\right)+{\text{w}}_{\alpha }^{T}\text{e}\left(t\right)=q_{\alpha }\left(t\right)+{\text{w}}_{\alpha }^{T}\text{e}\left(t\right)$$. [5]

In short, the beamformer estimated source strength,$\hat{q_{\alpha }}\left(t\right)$is equal to the true source strength,$q_{\alpha }(t)$plus the sensor noise projected through the beamformer weights. Substituting for the beamformer weights, this expression becomes

$$\hat{q_{\alpha }}\left(t\right)=q_{\alpha }\left(t\right)+\frac{{\text{L}}_{\alpha }^{T}{\text{C}}^{-1}\text{e}\left(t\right)}{{\text{L}}_{\alpha }^{T}{\text{C}}^{-1}{\text{L}}_{\alpha }}$$. [6]

In practice, the covariance matrix,C, is estimated from the MEG data, but for the purposes of our analytical model, it can be expressed mathematically as

C=〈bbT〉  =〈(Lαqα(t)+e(t))(Lαqα(t)+e(t))T〉≈Q2LαLαT+υ2I​, [7]

whereQis the standard deviation of the true source time course,υis the standard deviation of the sensor noise (which we assume to be equal for all sensors), andIis the identity matrix. The Sherman–Morrison formula allows us to compute the analytical form of the inverse of the covariance matrix, giving

$${\text{C}}^{-1}\approx \frac{1}{\nu^{2}}\left(\text{I}-f\frac{{\text{L}}_{\alpha }{\text{L}}_{\alpha }^{T}}{{\left\|{\text{L}}_{\alpha }\right\|}^{2}}\right)$$, [8]

where$f=\frac{Q^{2}{\left\|{\text{L}}_{\alpha }\right\|}^{2}}{\nu^{2}+Q^{2}{\left\|{\text{L}}_{\alpha }\right\|}^{2}}$is a scaled measurement of signal to noise ratio (SNR) and$\left\|{\text{L}}_{\alpha }\right\|$is the Frobenius norm of the forward field (Brookes et al., 2008). Substituting for${\text{C}}^{-1}$inEquation 6, it becomes simple (seeAppendix 1) to show that

$$\hat{q_{\alpha }}\left(t\right)=q_{\alpha }\left(t\right)+\frac{\upsilon }{{\left\|{\text{L}}_{\alpha }\right\|}^{2}}{\text{L}}_{\alpha }^{T}\varepsilon \left(\text{t}\right)$$. [9]

Note we have writtene(t)=νε(t), whereε(t)is a Gaussian random process (reflecting random sensor noise) with unit standard deviation.

Equation 9represents a single point in time, with the error between the true source time course,$q_{\alpha }\left(t\right)$and the reconstructed time course$\hat{q_{\alpha }}\left(t\right)$described by a stochastic process. However, a more useful representation of error would be a metric summed over time. The most obvious measure of total error is the square root of the sum of the squared errors. Mathematically,

Etot=1N∑i=1N(qα^(t)−qα(t))2=υN‖Lα‖2∑i=1N(LαTεi)2. [10]

Note the normalisation by the square root of the total number of timepoints (*N*) is to make this quantity equivalent to the standard deviation of the source projected noise. It now proves instructive to write the quantity${\text{L}}_{\alpha }^{T}\varepsilon_{i}$explicitly; if channel number is indexed byjthen we can say${\text{L}}_{\alpha }^{T}\varepsilon_{i}=\sum_{j=1}^{M}\;l_{j}\varepsilon_{ij}$(where$l_{j}$is the forward field for sensorj(i.e., the$j^{th}$element in${\text{L}}_{\;\alpha }$). It, therefore, follows that,

$$E_{tot}=\frac{\upsilon }{\sqrt{N}{\left\|{\text{L}}_{\;\alpha }\right\|}^{2}}\sqrt{\sum_{i=1}^{N}{\left(\sum_{j=1}^{M}\;l_{j}\varepsilon_{ij}\right)}^{2}}$$. [11]

The term${\left(\sum_{j=1}^{M}\;l_{j}\varepsilon_{ij}\right)}^{2}$is simplified considerably because$\varepsilon_{ij}$is a random process. That is, if we assume that there were only three sensors, so thatj=1,2,3, we can write,

(∑j=1Mljεij)2=(l1εi1)2+(l2εi2)2+(l3εi3)2+2l1εi1l2εi2 +2l1εi1l3εi3+2l2εi2l3εi3. [12]

Because$\varepsilon_{ij}$is random, and mean centred, while the${\left(l_{j}\varepsilon_{ij}\right)}^{2}$terms will always be positive, the cross-terms (e.g.,$2l_{1}\varepsilon_{i1}l_{3}\varepsilon_{i3}$) could be positive or negative, and because the process is stochastic, those terms will likely sum to zero. We can, therefore, write that${\left(\sum_{j=1}^{M}\;l_{j}\varepsilon_{ij}\right)}^{2}$≈$\sum_{j=1}^{M}\;l_{j}^{2}\varepsilon_{ij}^{2}$and it is convenient that, on average,$\varepsilon_{ij}^{2}=1$. This means that$\sum_{j=1}^{M}\;l_{j}^{2}\varepsilon_{ij}^{2}\approx \sum_{j=1}^{M}\;l_{j}^{2}={\left\|{\text{L}}_{\alpha }\right\|}^{2}$and so

$$E_{tot}=\frac{\upsilon }{\left\|{\text{L}}_{\alpha }\right\|}$$. [13]

The error in beamformer reconstruction is therefore directly proportional to the standard deviation of the sensor noise and inversely proportional to the Frobenius norm of the forward field.νis an invariant property of the sensors. However,$\left\|{\text{L}}_{\alpha }\right\|=\sqrt{\sum_{j=1}^{M}\;l_{j}^{2}}$and can be changed by system design (seeFig. 1). For example, if we increase the channel density,$\left\|{\text{L}}_{\alpha }\right\|$increases. Likewise, if we focus more channels in a region of high field,$\left\|{\text{L}}_{\alpha }\right\|$will again increase. The finding that noise falls with increasing channel count is not surprising since averaging signals across additional channels will always diminish noise. However, simple averaging would suggest that noise would drop as$1/\text{​}\sqrt{M}$, whereas our theory suggests a more drastic reduction, closer to1/​M, or conversely a linear scaling of SNR with$\left\|{\text{L}}_{\alpha }\right\|$, as a result of the beamformer model. If this scaling can be achieved in practice, then channel count and flexible array design will have a marked effect on the SNR of OPM-MEG.

**Fig. 1.**
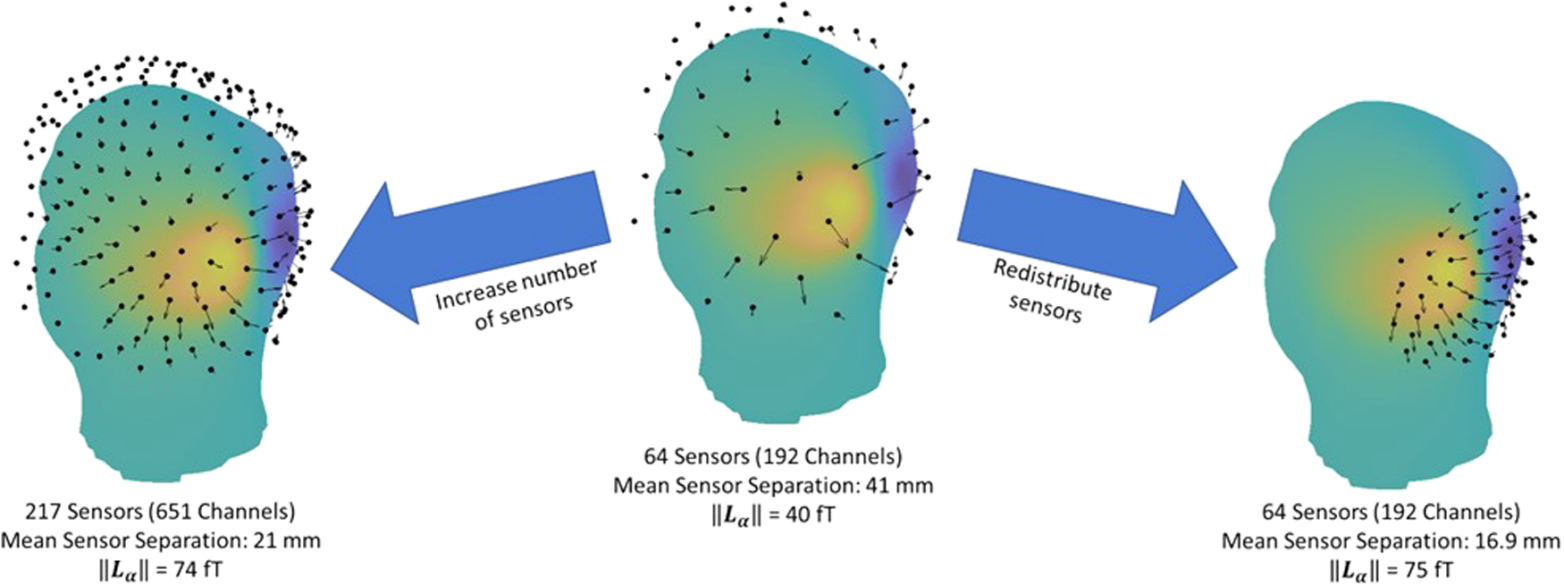
Changing sensor array to increase$\left\|{\text{L}}_{\alpha }\right\|$. The centre panel shows 64 sensors evenly distributed around the head. A source is placed in the occipital lobe and its forward fields are projected onto the surface of the head. The arrows show the magnitude and direction of the field measured at each sensor. The left-hand panel shows the effect of increasing the number of sensors on$\left\|{\text{L}}_{\alpha }\right\|$while the right-hand panel shows the effect of redistributing sensors to the region of high field.

## 3 Simulations

The above theory assumes a simple model with a single source, uncorrelated sensor noise, no interference, perfect co-registration of sensor locations to brain anatomy, and a perfectly estimated covariance matrix. In practice, these properties are likely to be unachievable. We, therefore, undertook a set of simulations.

### 3.1 Method

#### 3.1.1 Simulation 1: A single source with Gaussian noise

**Fig. 2.**
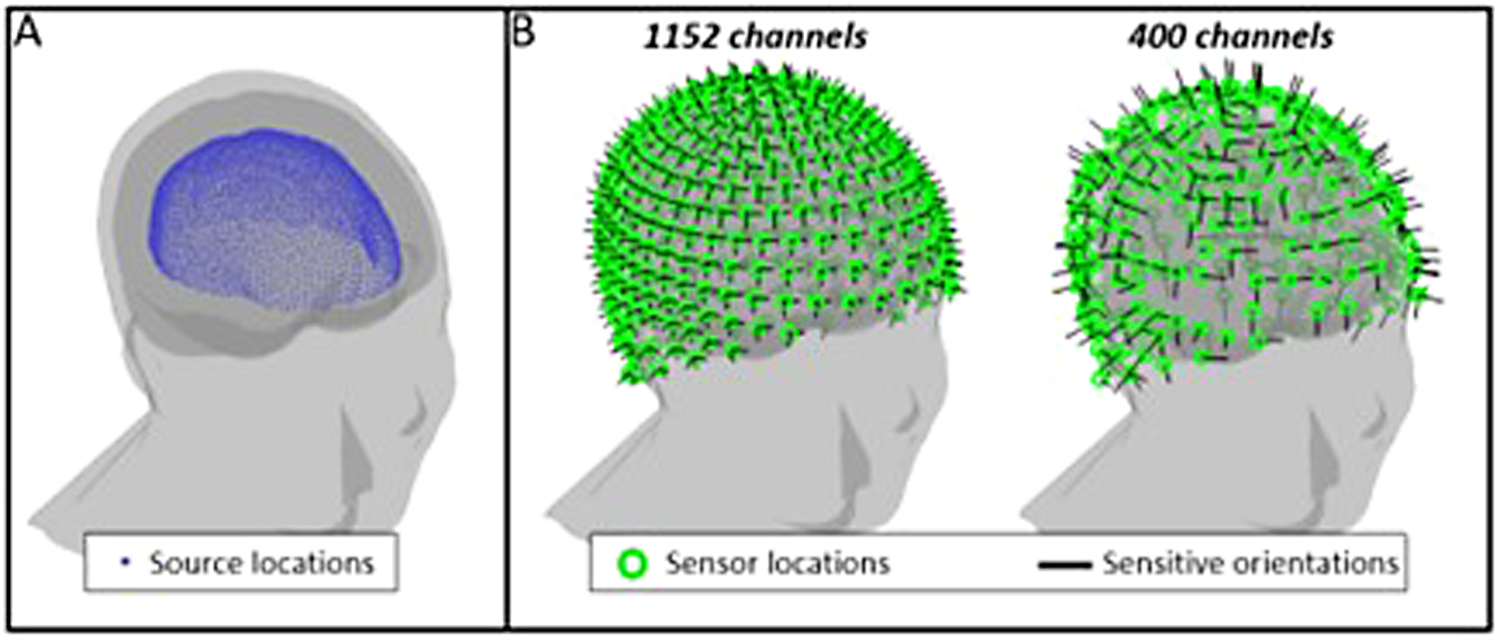
Schematic showing the simulation geometry. (A) Potential source locations (blue dots). (B) Sensor locations (green) and sensitive orientations (black). A total of 384 triaxial sensors were simulated, giving a maximum of 1152 channels (left). Reduced channel count was simulated by randomly removing individual channels. The example (right) shows 400 channels.

The purpose of this simulation was to verify our theory for a simple (single source) model. A single dipolar cortical source was simulated, with a depth of 2 cm from the surface of the brain. The location was selected at random from a total of 2944 possible locations (seeFig. 2A); the source orientation was randomised but constrained to the tangential plane, due to the relative insensitivity of MEG to radially oriented dipoles. A total of 384 sensor locations was simulated around the head with sensors uniformly spaced and positioned 6 mm above the scalp. Each sensor was assumed to have triaxial sensitivity (as is the case with current state-of-the-art OPMs (Shah et al., 2020)) (Fig. 2B, left panel). The forward field was computed using a single shell head model (Nolte, 2003) implemented in Fieldtrip (Oostenveld et al., 2011). The dipole time course comprised Gaussian random noise with a standard deviation of 1 nAm; 5-s periods of activity were interspersed with 5-s rest periods (where the amplitude was set to zero) to mimic trials in an experiment. A total of 300 s of data (30 trials) were simulated at a sampling frequency of 600 Hz. The source time course was projected through the forward field, and Gaussian random noise added to the resulting data with a standard deviation of either 20 fT, 40 fT, or 60 fT. To investigate how channel density affects overall sensitivity, the number of channels was varied, from 200 to 1100, in steps of 100. For each channel count, channels were selected at random (seeFig. 1B, right panel, for an example). Fifty iterations of the simulation were run for each channel count, and noise amplitude (with a different source location on each iteration).

For each iteration of the simulation, the data were reconstructed using a scalar beamformer (as perequations 2-4). Data covariance was estimated over all time and in the 1-300 Hz frequency range. We assumed no error in the forward field (which was again based on a dipole model and a single shell volume conductor). We constructed a pseudo-T-statistic for each of the potential source locations (the blue dots inFig. 2A), by contrasting source power during the active windows (when the dipole was switched on) to source power in the control window (when the dipole amplitude was set to zero). For all voxels, source orientation was calculated as that with the highest projected source power (Sekihara et al., 2004). We calculated the location of the highest pseudo-T-statistic and measured Euclidean distance to the true dipole location, as a measure of localisation error. For the peak location, we used the beamformer to reconstruct a time course of electrical activity, from which we derived two measures:

1) The square root of the sum of the squared differences between the reconstructed and the original (simulated) time course (i.e., equivalent to$E_{tot}$inEquation 13)
2) The SNR, which was defined as the ratio of the standard deviation of the reconstructed source activity, in the active and control windows (note here that during the control windows the dipole was switched off, and so any reconstructed signal variance is purely due to noise).

### 3.1.2 Simulation 2: Introducing realistic errors

To encapsulate more realistic scenarios, we simulated three different types of error:

1.**Brain sources of no interest:**In addition to the source of interest (which again had amplitude 1 nAm with 5 s of activity and 5 s rest, per trial), we simulated 100 additional sources in the brain. The source locations were chosen randomly from the 2944 possible locations inFigure 2A, but locations were a minimum of 1 cm from the source of interest. Source orientations were randomised but constrained to the local tangential plane. The interference source time courses comprised Gaussian random data, and source strengths were varied, taking values of 0%, 5%, and 10% of the standard deviation of the source of interest.

2.**External interference:**100 magnetic dipoles were simulated at random locations outside the head. Locations were between 15 cm and 50 cm from the centre of the head, but otherwise randomised, as were the dipole orientations. Dipole time courses comprised Gaussian random noise, whose amplitude was set such that the norm of the measured field at the sensor array was either 0%, 50%, or 100% of the norm of the field from the source of interest in the brain. These sources represent, for example, stimulation equipment in the MSR, patient implants (e.g., vagal nerve stimulator), or sources of no interest in the body (e.g., heart).

3.**Co-registration error:**In all the above simulations, we assumed that the sensor locations and orientations relative to brain anatomy were known accurately, and consequently the forward field used for the beamformer reconstruction accurately reflected the fields generated by the source—however, this is challenging in practice. We, therefore, simulated a co-registration error in which the sensor locations used in the reconstruction were rotated (relative to the centre of the head) compared to their true locations. Rotations were randomised, with a magnitude of either 0°, up to 2.2°, or up to 4.4°.

For each of the above three scenarios, we varied the sensor count between 200 and 1100 sensors (as previously) and reconstructed pseudo-T-statistical images as well as metrics of total error and SNR.

### 3.2 Results

**Fig. 3.**
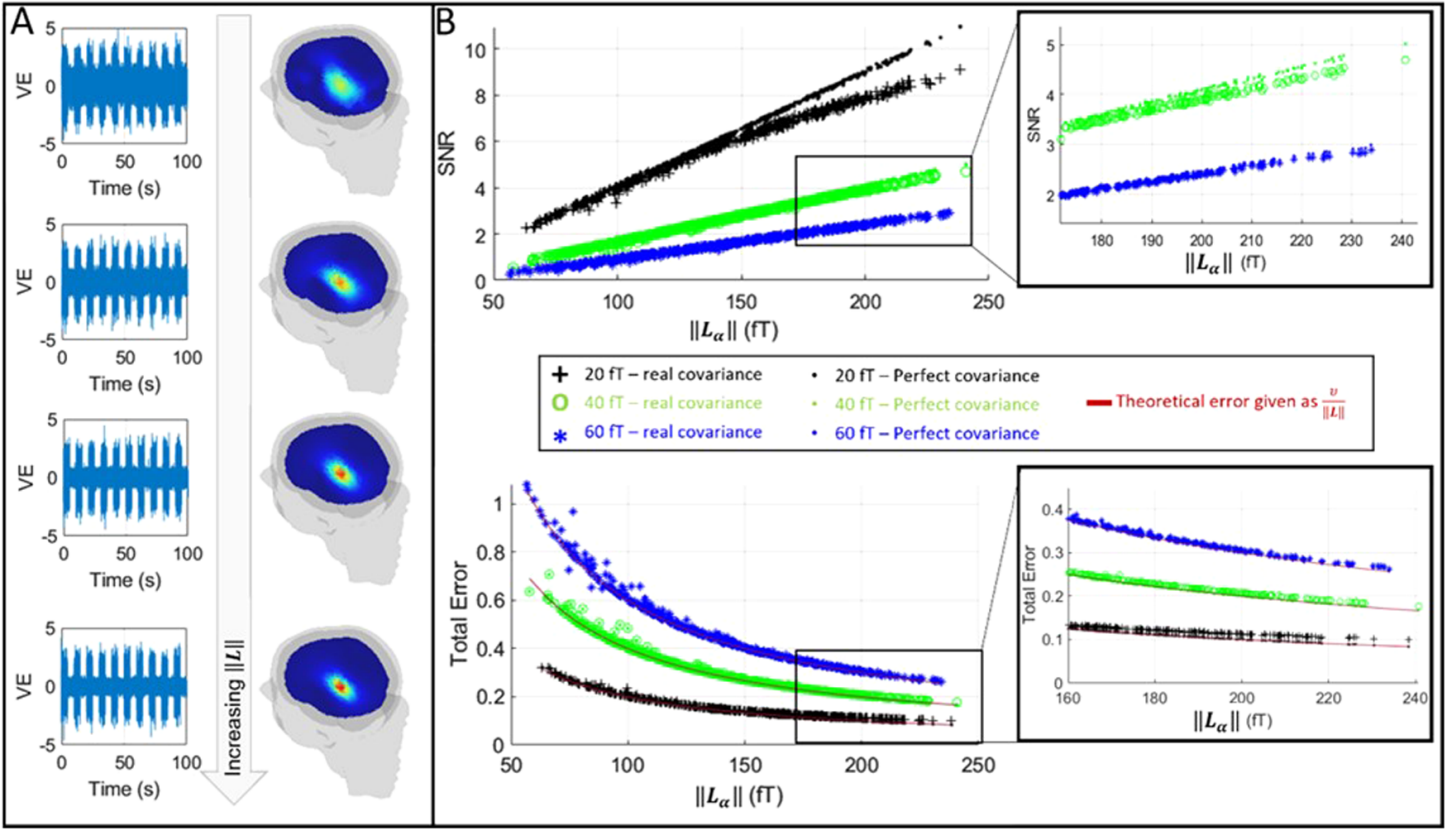
The relationship between SNR and total signal ($\left\|{\text{L}}_{\alpha }\right\|$): (A) Improving sensitivity. A single source of interest (amplitude 1 nAm) is simulated with gaussian sensor noise (amplitude 60 fT). Reconstructed source time courses (left) and beamformer images (right) shown for four different sensor counts (200, 500, 800, and 1100). (B) Improved SNR and decreased total reconstruction error with increasing$\left\|{\text{L}}_{\alpha }\right\|$: Upper plots show SNR; lower plots show total error in the time course reconstruction (i.e.,${\text{E}}_{\text{tot}}$). Three noise amplitudes are shown—20 fT (black), 40 fT (green), and 60 fT (blue). Dots show the case where an analytical form of the covariance matrix is used. Red lines show the theoretical result.

Figure 3shows results from Simulation 1.Figure 3Ashows the pseudo-T-statistical images and source time courses for channel counts of 200, 500, 800, and 1100. In the time courses, only the first 10 of 30 trials are shown. In all cases, the beamformer accurately delineated the source location; however, as channel count is increased, the pseudo-T-statistic grows stronger. In the reconstructed time courses, it is clear that the effect of sensor noise grows weaker with increasing channel count. These findings are formalised inFigure 3B, which shows SNR (upper panel) and total error (lower panel) plotted as a function of the total measured field (i.e.,$\left\|{\text{L}}_{\alpha }\right\|$). Blue stars show the case for a noise amplitude of 60 fT, green circles show a noise amplitude of 40 fT, and black crosses show a noise amplitude of 20 fT. In all three cases, coloured dots are also overlaid, and these represent the case where the covariance matrix has been formulated analytically (fromEquation 7) as distinct from data derived. In the case of the bottom plot, the red curve shows the analytical case fromEquation 13, which simply gives$\frac{\sqrt{N}\upsilon }{\left\|{\text{L}}_{\alpha }\right\|}$as a function of$\left\|{\text{L}}_{\alpha }\right\|$.

When using a perfect (analytical) covariance matrix, the simulation reproduced the expected theory with total error inversely proportional to$\left\|{\text{L}}_{\alpha }\right\|$and SNR scaling linearly with$\left\|{\text{L}}_{\alpha }\right\|$. In the case of data-derived covariance matrices, the data deviated slightly from the theory, and this was particularly noticeable at the low noise levels; specifically, the total error was marginally higher than theory would suggest, and the linear increase in SNR began to plateau at high channel count. This effect stems from sampling theory: previous work has shown that the error in the covariance matrix is directly proportional to the channel count (M), and inversely proportional to$\sqrt{N}$(whereNis the number of time points). This means that, as channel count increases, covariance error becomes larger unless compensated with longer recordings. A high covariance error has the effect of artificially diminishing beamformer reconstructed power, and it is for this reason that we see a tailing off of SNR as channel count is increased (Brookes et al., 2008).

**Fig. 4.**
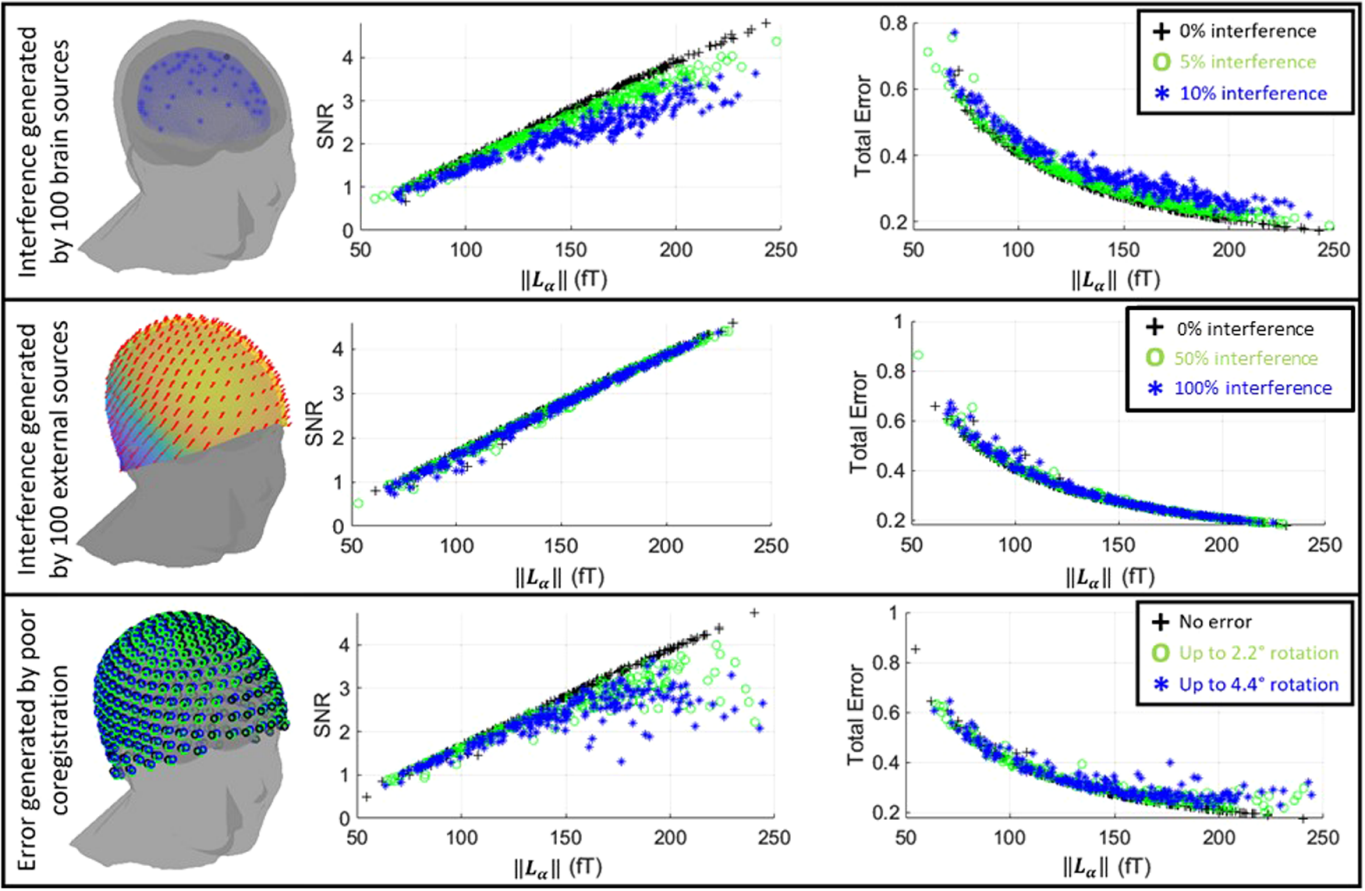
Top: The effect of interference from brain sources. A single source of interest is simulated alongside 100 interference sources. The left-hand plot shows an example, with the source of interest in black, and interference sources in blue. The relative amplitude of the interference was either 0% (black crosses), 5% (green circles), or 10% (blue stars) of the standard deviation of the source of interest. Middle: The effect of interference from external sources. The same source of interest is simulated alongside 100 additional magnetic dipoles outside the head. The left-hand plot shows an example field from a single interference source. The relative amplitude of the interference is either 0 (black crosses), 50% (green circles), or 100% (blue stars) of the norm of the field from the source of interest in the brain. Bottom: The effect of co-registration error. A single source of interest is reconstructed but error in the location and orientation of the sensor locations is added. Left-hand plot shows an example of helmet rotation; black crosses show no error; green circles show rotational errors up to 2.2 degrees; and blue stars show rotational co-registration errors up to 4.4 degrees. In all three cases, sensor noise was added with amplitude 40 fT/√Hz. The plots show SNR and total error versus$\left\|{\text{L}}_{\alpha }\right\|$.

Figure 4shows simulations incorporating realistic sources of error. In the upper plot, we include additional dipole sources in the brain; in the middle plot, we include external interference; and in the lower plot, we include co-registration error. In all three cases, an increase in$\left\|{\text{L}}_{\alpha }\right\|$continued to have a marked positive effect on SNR. External interference barely affected the SNR values—this is expected and highlights the ability of the triaxial array, coupled with beamforming, to spatially filter unwanted signals from distal sources (Brookes et al., 2021;Rea et al., 2022) (seeSupplementary Fig. S1for a radial-only comparison). Brain sources had a greater effect, though it is notable that SNR continued to increase, approximately linearly, with increasing$\left\|{\text{L}}_{\alpha }\right\|$. The largest effect came from co-registration error (which here can be generalised to any error in the model of the forward field). When large co-registration errors are simulated, the increase in SNR with increasing$\left\|{\text{L}}_{\alpha }\right\|$begins to plateau. This finding is in strong agreement with other recent simulations (Nugent et al., 2022) where forward field inaccuracy has a significant negative impact on the accuracy of OPM-MEG findings.

In summary, our simulations are in strong agreement with theory in showing an approximately linear improvement in SNR with$\left\|{\text{L}}_{\alpha }\right\|$. However, this is only the case when the forward field accuracy is high.

## 4 Experimental verification

### 4.1 Data acquisition

Our OPM-MEG system comprised 60 triaxial OPMs (QuSpin, Colorado, USA) integrated to form an array. Each OPM is a self-contained unit, containing a glass cell filled with^87^Rb vapour, a laser and associated optics for spin polarisation of the Rb atoms, and on-board electromagnetic coils for local field control across the cell. The mechanism by which these sensors measure magnetic field has been detailed extensively in previous publications (for reviews see,Schofield et al., 2023;Tierney et al., 2019) and will not be repeated here, save to say that each OPM enables measurement of three orthogonal components of magnetic field, with a noise floor of ~10-15 fT/√Hz (Boto et al., 2022). The sensors were housed in a magnetically shielded room (MSR), equipped with electromagnetic coils to control background magnetic field (Cerca Magnetics Limited, Nottingham, UK). Without the coils, the field inside the room is ~3 nT and stable over time in terms of magnitude and direction (Rea et al., 2022). To reduce this background field, the coils were energised using voltages that generate a field equal and opposite to that typically observed in the room. This has been shown to reduce background field to a level of ~0.7 nT (Rhodes et al., 2023). (Note—further application of nulling procedures can reduce this field (Rea et al., 2021,^2022^); however, this approach was not deemed necessary for this study). The sensors forming the OPM array were synchronised, data from each sensor were fed into a digital acquisition system and recorded by an acquisition computer. A separate (stimulus) computer controlled the experimental paradigms, including visual stimulation of the participant via a data projector (ViewSonic PX748-4K), which projected through a waveguide in the MSR and onto a back projection screen located inside. Stimulus timings were recorded via the addition of trigger channels, which placed markers in the data recorded by the acquisition PC. A schematic of the system is shown inFigure 5A.

**Fig. 5.**
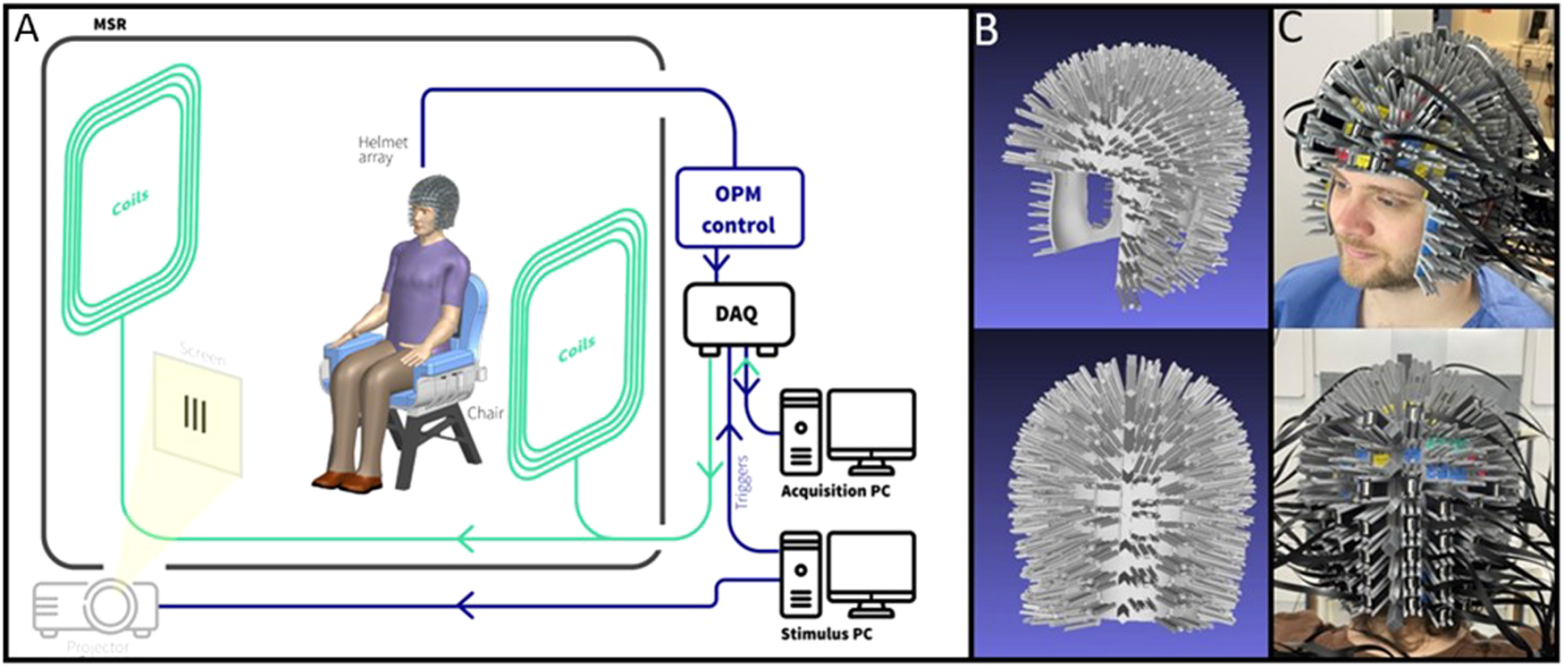
OPM-MEG system. (A) System schematic. (B) CAD illustration of a subject-specific helmet. (C) 3D printed helmet in place and housing triaxial OPM sensors. (Photographs shown with written, informed consent).

Our simulations (Fig. 4), in agreement with previous work (Nugent et al., 2022), demonstrate the importance of accurate knowledge of sensor locations and orientations relative to brain anatomy. To achieve this in practice, the OPMs were located on the scalp using a subject-specific helmet (Cerca Magnetics Limited, Nottingham, UK). Briefly, using an anatomical magnetic resonance image (MRI) (acquired using a Phillips Ingenia 3 T MRI system running an MPRAGE sequence, with 1 x 1 x 1 mm^3^resolution) we created a digital representation of the subject’s head shape, and used this alongside computer aided design (CAD) software to design a helmet that conforms to head shape, with sensor mounts on its outer surface (Fig. 5B). This helmet was then 3D printed (Fig. 5C). The process results in a digital representation of the helmet, in the same space as the MRI scan, meaning that sensor locations and orientations are known accurately relative to the brain anatomy.

The helmet contained 206 possible slots to house OPMs, however we had only a maximum of 60 OPMs (180 channels) available. An array was therefore designed where 30 of the sensors (90 channels) were packed as densely as possible, covering the occipital cortex. The locations of these sensors were based on a model in which we simulated a dipole in primary visual cortex (defined by the automated anatomical labelling atlas (AAL) (Tzourio-Mazoyer et al., 2002)) and calculated the locations at which the resulting (absolute) magnetic field would be a maximum. The remaining 30 sensors (90 channels) were placed equidistantly around the scalp (seeFig. 4C; the lower panel shows densely packed OPMs over occipital cortex, and the upper panel shows a frontal aspect where sensors were more sparsely distributed).

A single participant took part in the experiments. The participant gave written, informed consent prior to the experiment taking place, and all experimental protocols were approved by the University of Nottingham Faculty of Medicine and Health Sciences Research Ethics Committee. Two experimental paradigms were carried out; both aimed to elicit an increase in gamma band (>30 Hz) activity in the visual cortex.

- **Naturalistic visual stimulation.**A single trial comprised 5-s of naturalistic video footage, followed by a 5-s rest, during which a centrally presented fixation cross was shown. The footage comprised video of scenes, recorded by a head mounted camera, as an individual walked around a busy town (LONDON CITY TOUR, 2022). It was taken from YouTube, played in real time, and showed, for example, streets, roads, people, cars, and shops. The screen subtended a visual angle of 7° vertically and 12° horizontally. A total of 100 trials, each 10 s long, was recorded in a single experiment (meaning a total experimental duration of 1000 s). The experiment was repeated 6 times. The viewing of naturalistic images or video in this way has been shown to elicit broad band gamma band activity in animals (Kayser et al., 2003) and humans (Brunet & Fries, 2019). In most cases, these effects have been measured using iEEG, although there is also evidence that conventional MEG can measure similar phenomena (Chen & Farivar, 2020).
- **Moving circular grating.**A single trial comprised 2-s of visual stimulation, in the form of a centrally presented, inwardly moving, maximum-contrast circular grating. The grating subtended a visual angle of 5°. Stimulation was followed by a 3-s baseline period during which a fixation cross was shown in the centre of the screen. A total of 60 trials was used, giving a duration for a single experiment of 300 s. Again, the experiment was repeated 6 times. This paradigm has been well studied using MEG and is known to increase narrow band gamma oscillations (Hoogenboom et al., 2006;Iivanainen et al., 2020).

We deliberately chose paradigms that would elicit gamma band effects, since these phenomena are known to be low amplitude, and are therefore challenging to detect. In all cases, OPM-MEG data were recorded at a sample rate of 1200 Hz. For the naturalistic viewing paradigm, data collection was also repeated (with six experimental repeats, and an interleaved design) using conventional MEG (seeAppendix 2).

### 4.2 Data analysis

Following collection, data were bandpass filtered between 1 and 150 Hz. Channels with a noise floor greater than 30 fT/√Hz or less than 7 fT/√Hz in the 60 – 80 Hz range were marked as bad and removed. In all runs, three channels (one sensor) were consistently >30 fT/√Hz, while the remaining channels had an average noise floor of 12.7 ± 0.3 fT/√Hz in the 60 – 80 Hz range across all experimental runs. Bad trials, defined as those in which the standard deviation of the signal at any one sensor was greater than 4 times the average standard deviation of the signal at that sensor across all trials, were removed. Visual inspection confirmed this algorithm was successful. On average, it resulted in the removal of 6.5 ± 5.3 trials (mean ± std across all datasets).

Following pre-processing, data were projected to source space using a scalar beamformer. The beamforming methodology was equivalent for both tasks. A data covariance matrix was computed in the 52-65 Hz frequency band and was regularised using the Tikhonov method with a regularisation parameter equal to 0.01 times the maximum eigenvalue of the unregularised matrix. The forward field was based on a dipolar source and a single shell volume conductor model. The brain was divided into regular 4-mm voxels, and source orientation was taken as that with the largest projected signal. We derived a pseudo-T-statistical functional image which contrasted gamma power in active and control windows. For the naturalistic viewing task, the active window was 2 s < t < 4 s (where t represents time, relative to trial onset) and the control window was 7 s < t < 9 s. For the moving grating, the active window was 0.2 s < t < 1.8 s and the control window was 2.8 s < t < 4.4 s. For each experimental run and each task, we used 177 of the available 180 channels to derive a beamformer image to localise the source of maximum task induced gamma band modulation. Three channels were switched off due to high noise levels.

Based on the peak of the image, we computed a broad band (1-150 Hz) time course of electrophysiological activity (using a beamformer, in which the covariance matrix was also derived from broad band data). We also computed a narrow band time course (using 52-65 Hz derived covariance). We used the broad band time course to generate a time frequency spectrum (TFS), constructed by sequentially filtering signals into overlapping frequency bands, computing the envelope of oscillatory power using a Hilbert transform, averaging the envelope over trials, and concatenating the result in the frequency dimension. For the narrow band time course, we derived the envelope of activity using the Hilbert transform and averaged this across trials to create a single trial averaged response showing the modulation of gamma activity with time. From this, we derived a quantitative measure of SNR, as the mean signal during stimulation, minus the mean signal at rest, divided by the standard deviation of the signal at rest. For naturalistic viewing, the stimulation window was 0.5 s < t < 4.5 s and the rest window was 5.5 s < t < 9.5 s; for the grating task, the stimulation window was 0.2 s < t < 1.8 s and the rest window was 2.8 s < t < 4.4 s.

Our aim was to investigate how SNR changes with $\left\|{\text{L}}_{\alpha }\right\|$ . To this end, we first averaged the pseudo-T-statistical images across all experimental runs to get a single estimate of the location and orientation of the gamma source. Based on this, we used a dipole model to generate an estimated forward field $\left(\left\|{\text{L}}_{\alpha }\right\|\right)$ at all sensors, and the Frobenius norm gave an experimental estimate of $\left\|{\text{L}}_{\alpha }\right\|$ . We undertook two analyses:

- **Changing the channel count**: To investigate how channel density affects SNR, random channels in the 177-channel array were “turned off” during the analysis. The number of channels turned off ranged from 1 to 100, in steps of 10, with 100 iterations of random channel distributions for each channel count. We then used the beamformer to reconstruct the signal at the source location, and remeasured SNR. Both by reducing the channel count, and by switching off a different channel subset (for the same count) we were able to vary$\left\|{\text{L}}_{\alpha }\right\|$.
- **Contrasting a dense and sparse array**: The available channels were separated into two arrays: a**sparse**array comprising 30 sensors (90 channels) distributed uniformly across the head; and a**dense**array, also comprising 30 sensors (90 channels) packed close to visual cortex (see also above). This is an experimental realisation of the technique termed “magnetocorticography” originally reported byNugent et al. (2022), and it mimics a situation in which a 30-sensor array was redeployed to focus on a specific region—however, it does so in a single experiment to avoid confounds associated with subject performance. We measured both the SNR change in the beamformer-reconstructed source, and the effective change in$\left\|{\text{L}}_{\alpha }\right\|$, for both arrays.

These two analyses were carried out for every experimental run in both tasks, independently. They enabled experimental verification of our theoretical and simulated findings, as well as a practical demonstration of the effect on SNR of redistributing the OPM array.

### 4.3 Results

**Fig. 6.**
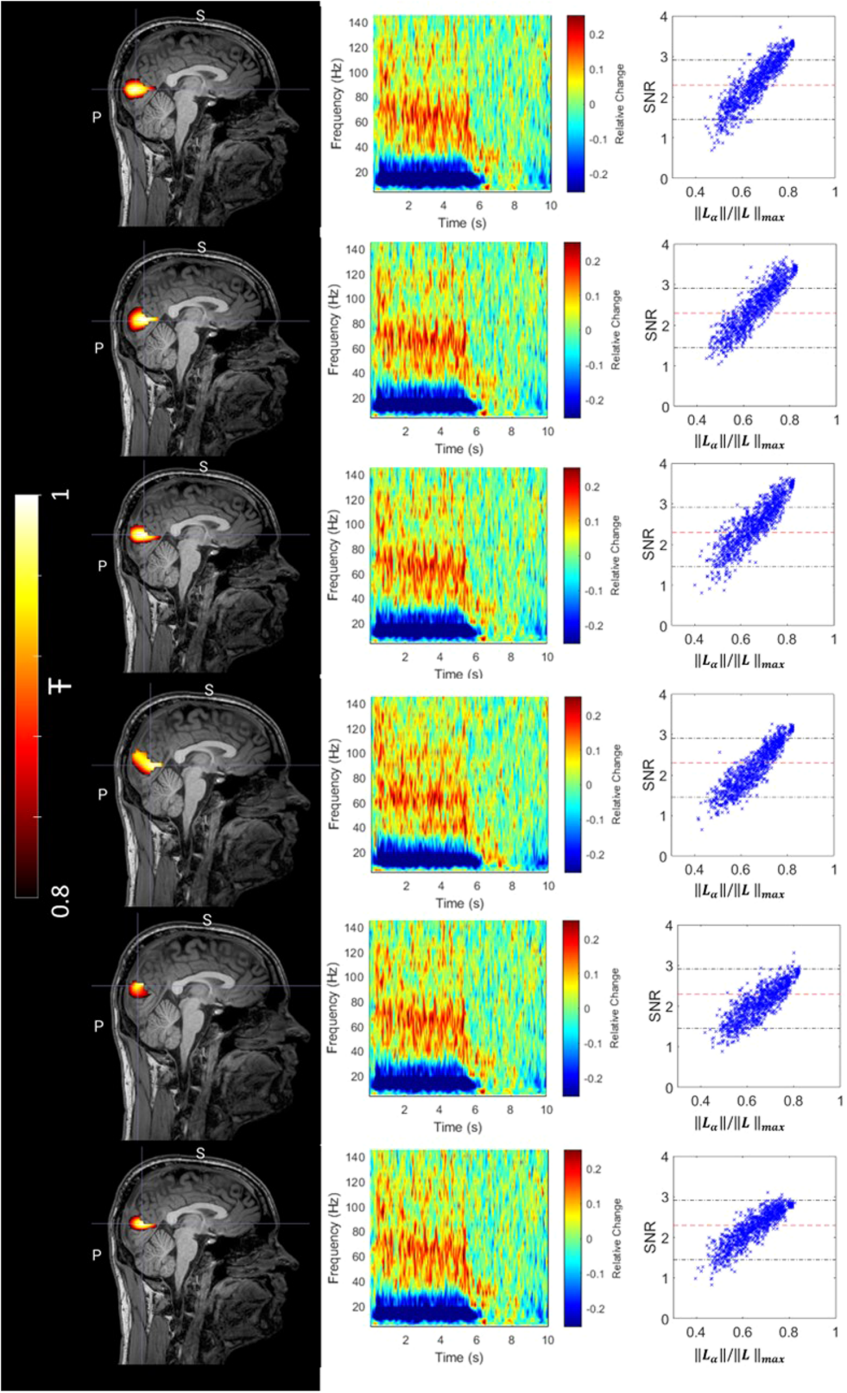
Results from the naturalistic viewing task. The separate rows show six independent experimental repeats. The left-hand panel shows the pseudo-T-statistical image depicting maximum regions of gamma modulation, overlaid onto a subject specific anatomical MRI. The functional image was thresholded at 80% of its maximum. The centre panels show a TFS, extracted from the average peak location (across runs). The right-hand panels show SNR plotted as a function of$\left\|{\text{L}}_{\alpha }\right\|$. Here,$\left\|{\text{L}}_{\alpha }\right\|$was altered by randomly removing different channels. Blue dots represent each iteration of random sensor array designs, and the red dashed and black dotted-dashed lines show the mean and range of the SNR values obtained using cryogenic MEG respectively. An equivalent figure for the grating experiment is shown inSupplementary Figure S2.

Figure 6shows the results from the naturalistic viewing task. Data from all six runs are shown independently in each row. For each run, the left-hand panel shows the pseudo-T-statistical image of gamma modulation, derived using all sensors and thresholded at 80% of its maximum. The central panel shows a TFS, again derived using all sensors. The TFS’s for all six runs are extracted from a single location in the brain, which was taken as the average peak location of all six runs (i.e., the average of the six images shown in the left panel). The right-hand column shows how SNR varies with$\left\|{\text{L}}_{\alpha }\right\|$. Values were normalised by dividing by the highest possible value of$\left\|{\text{L}}_{\alpha }\right\|$which would be achieved if all 206 sensor slots in the subject specific head cast were populated with triaxial sensors. Note that lower values of$\left\|{\text{L}}_{\alpha }\right\|$mean either fewer channels were used, or that the channels in use were not located over areas of high signal; values close to one mean that we are close to detecting the maximum possible signal allowed by the helmet layout. Each point in the scatter plot represents either a different channel count, or a different (random) selection of channels used for the reconstruction.

The peak in gamma modulation was located within the primary visual cortex as would be expected. The coordinate at which the largest modulation was observed was [-1 ± 3, -71 ± 7, 36 ± 6] mm (mean ± standard deviation across runs). The Euclidean distance between the mean source location across runs and any single experiment was 8 ± 4 mm. This demonstrates that source localisation across separate experimental runs was stable—likely a consequence of the subject specific helmet which ensures consistent sensor placement for each use.

The TFSs from primary visual cortex show a strong broadband gamma response—this is very similar to that observed previously using iEEG in both animals (Kayser et al., 2003) and humans (Brunet & Fries, 2019) (yet is markedly different to the narrow band response observed for the grating stimulus—see below inFig. 7).

**Fig. 7.**
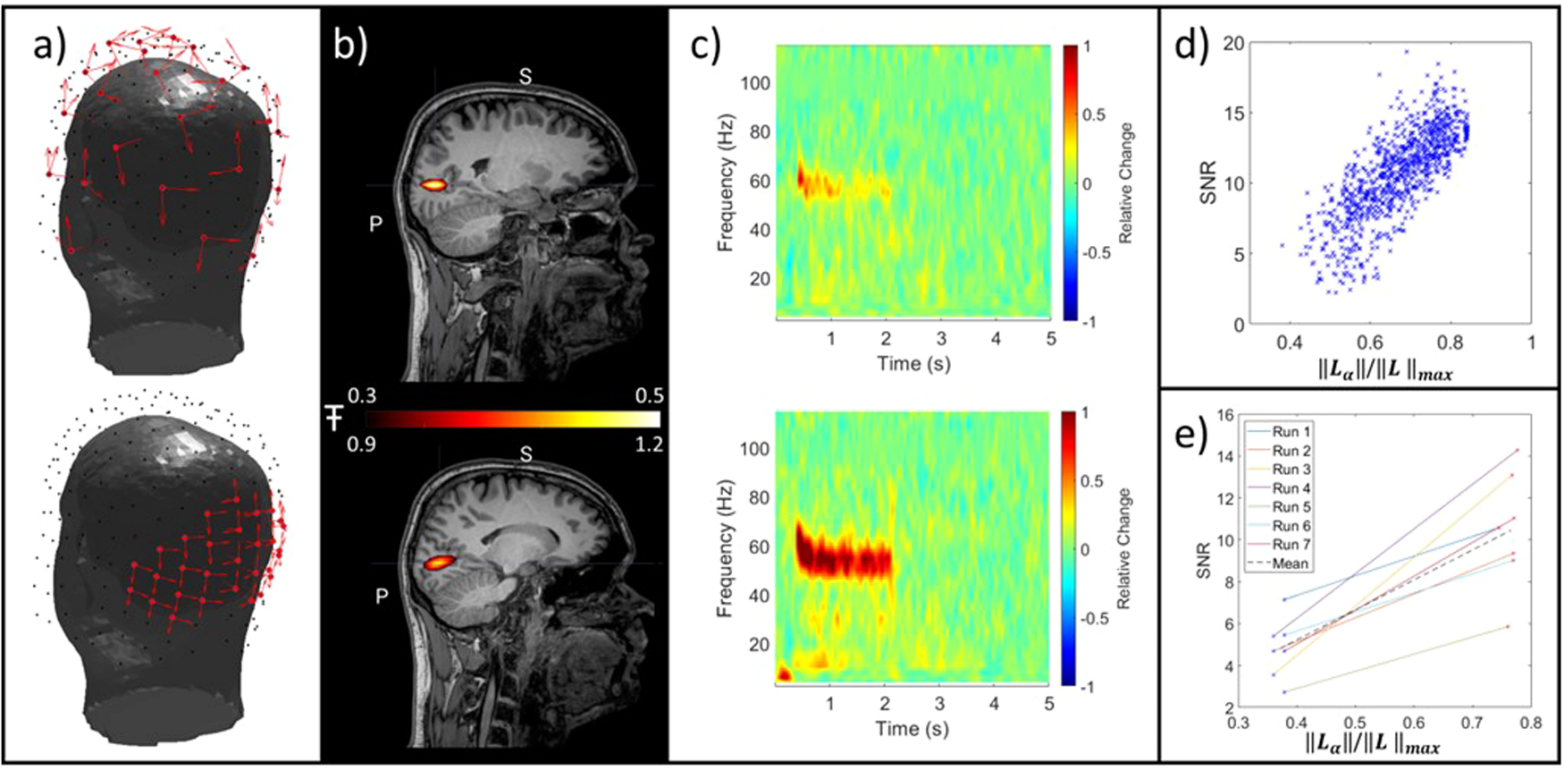
Results from the grating task. (A) Triaxial channel locations and orientations for the sparse and dense array. Both arrays had a total of 30 sensors (90 channels). (B) Pseudo-T-statistical images showing the spatial signature of the largest gamma modulation. Average across all runs is shown (C) TFSs extracted from the location of peak gamma change. Plots show a single example run. In all the above cases, the upper plot shows the sparse array, and the lower plot shows the dense array. A single example run is shown. (D) SNR plotted as a function of$\left\|{\text{L}}_{\alpha }\right\|$for a single run—again, note the approximately linear dependency. (E) SNR improvement (for all six runs) by moving from the sparse to the dense array.

Most notably, the plots in the right-hand panel show that SNR increases approximately linearly with$\left\|{\text{L}}_{\alpha }\right\|$. This agrees with both our theoretical findings and our simulations. For non-optimal array designs (low$\left\|{\text{L}}_{\alpha }\right\|$) SNR was ~1; however, this was typically increased to >3 when$\left\|{\text{L}}_{\alpha }\right\|$was higher. The pink bar shows, for comparison, the SNR values that were attained from similar experiments using a SQUID system (seeAppendix 2). The mean SNR for the OPMs (using all channels) was 3.0 ± 0.2; the equivalent SNR for the SQUIDs was significantly (p = 0.013, Wilcoxon sum rank test) lower, at 2.3 ± 0.5. These values provide a useful benchmark for where we might expect SNR to be using current MEG technology.

Figure 7shows results from the visual grating task. Here, we have contrasted the two separate 90-channel arrays, one where sensors are distributed equally across the scalp and one when they are clustered over the region of interest. Note that the data from the two arrays were recorded concurrently (to avoid confounds that the brain may behave differently were it recorded across two runs). Nevertheless, this mimics a situation where one only has 30 sensors, and they are redistributed over an area of interest.

Figure 7Ashows the locations and orientations of the sensors for the two arrays. The black dots show all possible slots on the subject specific helmet; the red arrows show the channel locations and orientations that were used for each array.Figure 7Bshows the pseudo-T-statistical images for the two arrays (averaged across runs); on average, the two arrays demonstrated similar localisation; for the sparse array the coordinate with the highest pseudo-T-statistic was at [-13 ± 8, -73 ± 2, 30 ± 4] mm (mean ± standard deviation across runs) and for the dense array it was at [-15 ± 2, -74 ± 2, 29 ± 2]. However, the peak Pseudo-T-statistic itself fell from 1.14 ± 0.14 (dense array) to 0.43 ± 0.15 (sparse array).Figure 7Cshows representative TFS’s generated at the location of highest gamma modulation for a single representative experimental run. As expected from previous work (e.g.,Hoogenboom et al., 2006) (yet distinct from naturalistic viewing), a narrow band gamma response lasting the duration of visual stimulation is clear. This is observed for both arrays but is clearer in the dense array. This finding is formalised inFigures 7Dand7E;Figure 7Dshows SNR plotted against$\left\|{\text{L}}_{\alpha }\right\|$for a single representative experimental run, once again the approximate linear trend of improvement in SNR is observed.Figure 7Eshows the SNR improvement between the sparse and dense arrays for all six runs (the lines join the SNR values for each experiment). Between the two arrays,$\left\|{\text{L}}_{\alpha }\right\|$increased by a factor of 2.1, whilst the mean SNR went from 4.8 ± 1.4 to 10.5 ± 2.8, a factor of 2.2.

## 5 Discussion

Our results have shown that increasing the total signal measured by an OPM-array$\left(\left\|{\text{L}}_{\alpha }\right\|\right)$affords a linear proportional increase in the SNR of a beamformer projected source estimate. Manipulating the total measured signal can be achieved by either increasing the total number of channels (e.g., by adding more OPMs to an array) by using triaxial rather than dual or single axis OPMs, or by redistributing a fixed number of OPMs to better cover the scalp locations where the source of interest generates the largest field. The rapid (linear) increase in SNR with total signal was predicted by beamformer theory, confirmed by simulation, and further confirmed by experimental measures of narrow and broad band gamma effects elicited by visual stimulation. These findings demonstrate the advantages of the flexibility of OPM-MEG (distinct from conventional MEG) to redistribute sensors in an array to focus on a specific region (Nugent et al., 2022). They also show experimental evidence of the high importance of expanding OPM-MEG systems to higher channel count (which has been highlighted by previous theory papers (e.g.,Boto et al., 2016;Iivanainen et al., 2017).

Our theoretical (analytical) analysis comprised a generative model, with a single dipolar current source contaminated with additive Gaussian sensor noise. This is a simple model, but it has nevertheless proved instructive for optimising beamformer performance (e.g.,Brookes et al., 2008). The outcome was a finding that total error in a beamformer reconstruction should fall as$1/\left\|{\text{L}}_{\alpha }\right\|$, or conversely that SNR should increase linearly with total signal. The result that SNR should increase with an increasing number of sensors is unsurprising; the more sensors present, the more signals we can average, and therefore the more the sensor noise will diminish^†^. However, if this were only due to averaging, one would expect that SNR should improve with the square root of sensor count, whereas this theory suggests it scales linearly. This can therefore be taken as a significant advantage of beamformer reconstruction.

Our simulations validated our theory, with total error in a beamformer reconstruction matching perfectly the analytical findings, in the case where the data covariance was free from error. In practice, our simulations showed that a perfect linear increase in SNR, in all cases, is unachievable; while it may look linear in certain regimes, data derived error in the covariance matrix increases with channel count and this is known to artefactually reduce beamformer projected power, and consequently cause a (slight) plateauing of SNR. This effect was most noticeable at low noise levels. The addition of dipole sources of no interest in the brain reduced the slope of the improvement (though it was still approximately linear) while the addition of external interference had little effect (a result of the excellent interference suppression properties of a triaxial array design (Brookes et al., 2021;Rea et al., 2022;Tierney et al., 2021)). By far the largest effect on improvement in SNR with total signal however came about due to co-registration error, which caused a marked plateauing of SNR. Importantly, although we simulated error in co-registration, this result can be generalised to any error in the forward field, which could be due to OPM gain errors, orientation errors, cross-talk, or inaccuracies in the dipole approximation or volume conductor models. Thus, in strong agreement with previous work (Nugent et al., 2022), the improvements afforded by high sensor density (or judicious placement of OPMs) are only realisable in practice if the modelled forward field is of high accuracy.

When we measured how SNR varies with total signal in an experimental setting, the improvement was indistinguishable from linear, and this has significant implications for the way in which we undertake OPM-MEG array design. Despite their high practicality and utility, OPMs still have a higher inherent noise compared to a SQUID—perhaps by a factor of 3-6 (depending on how the OPM is configured and the type of SQUID used). The lack of requirement for cryogenic cooling, however, means that OPMs get closer to the head surface, picking up a 4 to 5 times larger signal (for cortical sources). These two effects, at least in theory, cancel out leaving OPM and conventional systems approximately equal in terms of SNR for cortical sources (e.g.,Hill et al., 2020;Rhodes et al., 2023). Here, our results show that the higher noise level of OPMs can be compensated, either by increasing channel count, or by exploiting flexibility of sensor placement. This is an important finding, since it shows that while the ongoing work to reduce inherent sensor noise remains of extremely high importance, assuming sensors have a small footprint they can by packed in a high-density array to achieve very high sensitivities even despite their noise floor. It is worth noting that triaxial sensitivity also provides a significant advantage: It is well known (Iivanainen et al., 2017) that the two tangential components of magnetic fields generated by the brain are smaller than the radial field. Nevertheless, triaxial measurement approximately doubles the total signal recorded. This, in turn, increases the SNR of a beamformer reconstructed source. This is notwithstanding the significant enhancements in interference rejection which are also afforded by triaxial measurement. So, in sum, by a combination of triaxial measurement and high sensor density, it should be possible for an OPM-MEG system to outperform significantly the current state-of-the-art MEG instrumentation, in terms of sensitivity.

In a practical sense, it is noteworthy that the redistribution of sensors to a specific area of the scalp requires an a-priori assumption about which brain area to target. In many cases this is possible—for example, localisation of epileptogenic foci where EEG or MRI have provided target regions, or in paradigms that target specific regions. However, a-priori assumptions are not available generally—particularly in studies of distributed networks. In some cases, it may be possible to use a sparse (whole head) array to detect an effect and then a dense array to optimise SNR—“zooming in” on a single network node. Ultimately however, our results highlight a pressing need to increase the total number of OPMs in MEG arrays. Increasing sensor density without compromising coverage is currently costly, but an active area of research.

**Fig. 8.**
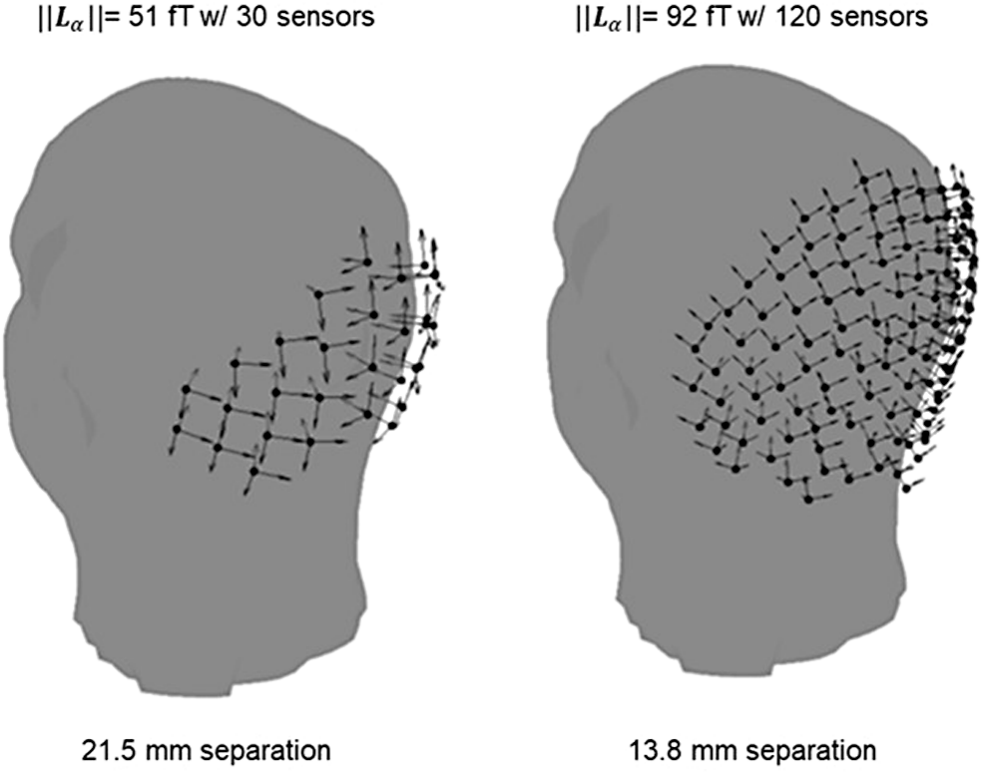
Potential OPM array. (Left) The current 30-sensor (90-channel) dense sensor array in the bespoke helmet. (Right) A hypothetical (but achievable) 120-sensor (360-channel) array with an average sensor separation of 13.8 mm. For reference, a QuSpin Gen-3 OPM has a footprint of 12.4 x 16.6 mm.

It was not our direct intention to compare conventional- and OPM-MEG SNR within this paper. Nevertheless, to provide an indicative benchmark for SNR we ran similar experiments, in the same subject, using a conventional MEG system. The result showed that our OPM-array—using all 177 channels (many of which were focussed over the visual areas)—offered an SNR that was 1.3 times greater than our SQUID system. This result needs to be treated with a great deal of caution: We set up the experiments in as similar a way as possible (e.g., we used the same subject, interleaved experiments to minimise habituation effects, we used the same stimulus with a matched visual angle). However, other factors may induce differences. For example, the ambient lighting inside the two MSRs was slightly different; the projectors used were different, the viewing angle was also different; and the co-registration procedure was different for each system and so this may lead to forward model errors. These differences were out of our control but could significantly influence SNR. More importantly, the SNR of our OPM system was limited by both the number of sensors we had available, and by the sensor density on the subject specific helmet, both of which could be increased significantly. Indeed, further simulations (Fig. 8) showed that, even with the current size of OPMs, we could easily increase the total measured signal from 54 fT (in our current 90-channel array) to 92 fT in a hypothetical 360-channel array. If SNR remains approximately linear with total signal, this likely means an SNR of ~5.1 is achievable, a factor of 2.2 larger than what we measured from our SQUID array. This could be increased even further by the design of smaller OPMs; while most OPM manufacturers are pursuing higher sensitivity, our work suggests a second avenue to explore would be to decrease the footprint to allow higher density arrays, perhaps employing solutions that use multiple cells within a single sensor head, enabling multiple measures over a very small area (Borna et al., 2020;Pratt et al., 2021).

One technical issue that warrants discussion is “crosstalk” (the potential for a systematic error in magnetic field measurement at one OPM, caused by the presence of a second OPM in close proximity). This is something that affects all OPM-MEG studies but is particularly important in high-density arrays, as sensors are sited closer together. Crosstalk arises because OPMs use on-board-sensor coils to generate a modulation field, which gives the sensor its directional sensitivity (see e.g.,Schofield et al., 2023;Tierney et al., 2019, for reviews on how this works). Critically, all OPMs within an array are fed with a coherent modulation field meaning that if two sensors (A and B) are close, the field in the cell of sensor A is the summation of the field from the coils of sensor A, and the stray field from sensor B. If the stray field is large, this can result in changes in sensor gain and/or sensitive orientation. Here, we deal with this problem via calibration. Specifically, three separate pulses of magnetic field are applied using the on-board sensor coils; any unwanted signal (e.g., generated on the y-axis measurement, from an x-axis pulse) can then be characterised, and corrected. This orthogonalisation—which is a unique benefit of triaxial sensors—means that the sensor will provide a true estimation of field magnitude and direction. This calibration scheme has proved successful—for example, a recent study (Rier et al., 2022) showed that when assessing fields from a phantom, the measured field from an array of triaxial sensors demonstrated a ground truth correlation of 0.99. Nevertheless, triaxial arrays of this type remain nascent technology and the calibration routine we use is only as good as the on-board sensor coils; any imperfections in the manufacturing process could re-introduce crosstalk errors. For this reason, further work on this important topic is required—particularly before OPM-MEG is used for clinical studies.

There are some limitations of this study which warrant discussion. First, our experimental results are limited to visual gamma oscillations. This means that 1) the signal is generated by a single dominant source in visual cortex and 2) interference from other brain activity in this frequency range is of low amplitude and so sensor noise is most likely to dominate over noise from other brain regions (this is less likely to be true in other frequency bands). Whether the linearity between SNR and$\left\|{\text{L}}_{\alpha }\right\|$is maintained when there are multiple sources warrants further experimental investigation, though our simulations inFigure 4suggest that this will be the case. Whether the linear relationship remains for lower frequency is a more open question: At lower frequency, signals from both the source of interest and elsewhere in the brain are larger, and the effect of sensor noise is comparatively smaller. It is therefore possible that for lower frequencies, the effect of high-density sensor layout on spatial resolution will be more important than suppression of sensor noise. Future work should therefore probe this experimentally. Second, here, we focus only on a beamformer approach to source localisation; it is important to note that the usual limitations of beamforming still apply—for example, suppression of highly correlated sources (such as those generated in the left and right auditory cortices by coherent binaural stimulation)—and more importantly that our findings may not translate to other inverse methods (e.g., minimum norm estimation). Mathematically speaking, MNE is similar to beamforming but the covariance matrix is replaced by a formulation based on the forward fields (Sekihara & Nagarajan, 2008). While different, these two matrices have a similar mathematical form and so it is likely that the relationship between SNR and$\left\|{\text{L}}_{\alpha }\right\|$might be maintained for MNE, and potentially other inverse solutions. However, verification of this would require analytical, simulation, and experimental work and so it is beyond the scope of the current study. Finally, our simulations showed the critical importance of accurate co-registration of sensor geometry to brain anatomy; as a result, we chose to undertake experimental measures using a subject-specific helmet to optimise co-registration accuracy. Whether similar linear improvement in SNR with total signal could be experimentally delineated in a case where, for example, a generic OPM helmet was used and co-registered using optical methods, remains an open question. This should be the object of future study, and if co-registration error does prove to diminish the advantages, then this provides motivation for new and more advanced co-registration techniques. Related, we also know that SNR improvement is not only affected by co-registration error but also by any error in the forward model. Future work may therefore benefit from more accurate head models (e.g., boundary or finite element models).

## 6 Conclusion

In non-invasive evaluation of human brain electrophysiology, achieving high sensitivity (to match that of invasive techniques) has proved challenging. OPMs offer a new opportunity in this area, yet their inherent noise floor remains high. Here, we investigate how judicious array design might increase sensitivity. Through theoretical analyses, simulation, and experiment, we have shown that increasing the total signal measured by an OPM-array (either by increasing the number of channels, or by changing the placement of OPMs) affords an approximately linear increase in the SNR of a beamformer projected source estimate. We used this to facilitate high sensitivity measurements of gamma band activity in the visual cortex that compared favourably to similar measures made using a conventional MEG system. These findings add weight to an increasing argument that OPMs are the sensor of choice for MEG system construction, they highlight the advantages of a system with flexible sensor placement, and most importantly they point to how, in the future, non-invasive techniques might be optimised to provide extremely high sensitivity measurements of human brain electrophysiology.

## Supplementary Material

Supplementary Material

## Figures and Tables

**Fig. A1.**
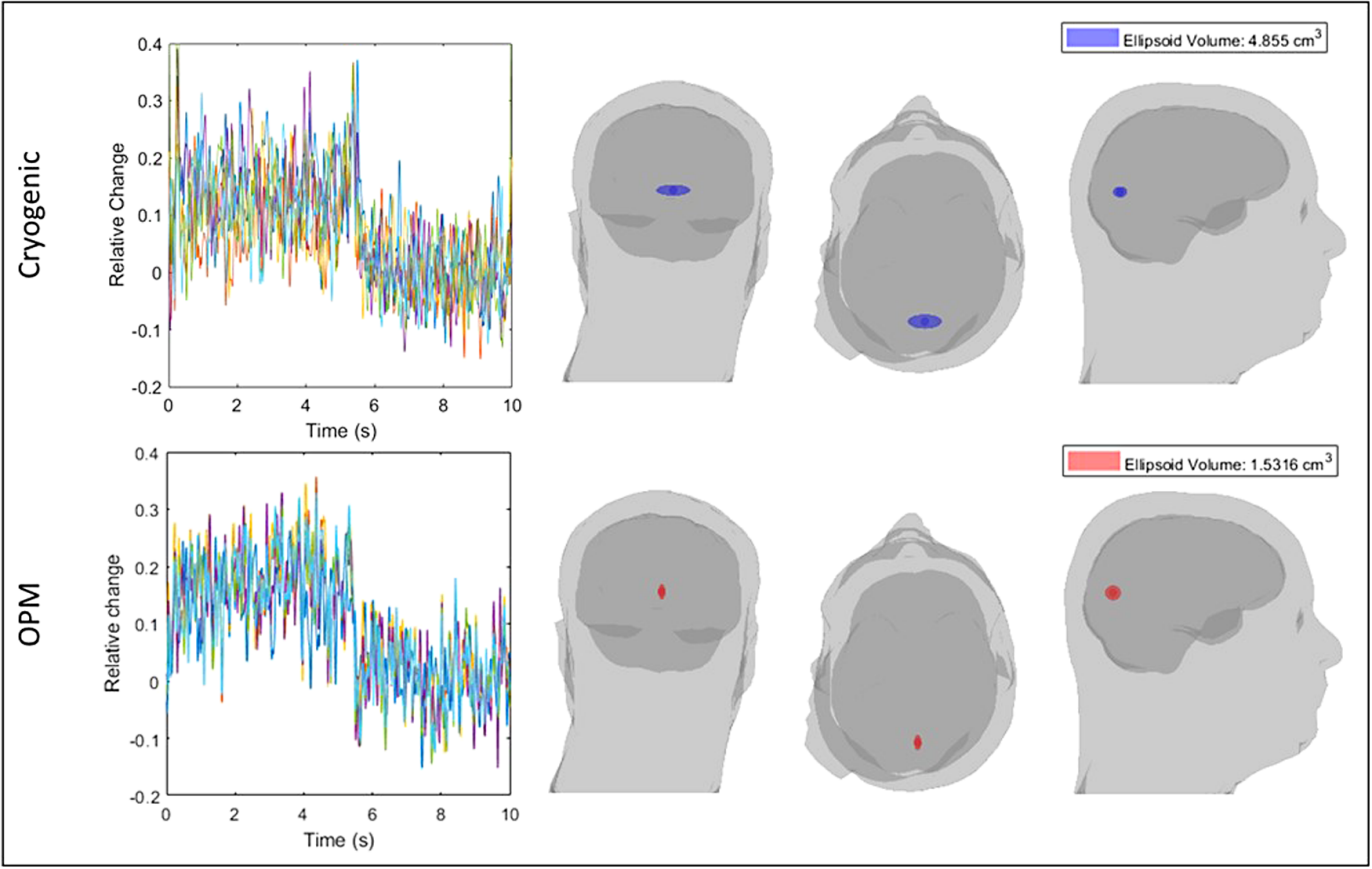
A cryogenic versus OPM-MEG comparison. The upper panel shows the cryogenic MEG, the lower panel shows the OPMs. In both cases, the line plots show gamma band envelopes. In the right-hand images, the ellipsoid shows the spread of peak locations identified from the pseudo-T-statistical images.

## Data Availability

All data were acquired by the authors and are available upon request. All code was custom developed in-house using MATLAB and is available from the authors on request.

## Footnotes

†
Note that this assumes that sensor noise is uncorrelated across channels; this is likely the case for MEG metrics in the gamma band but may not be the case for lower frequencies (which may be contaminated by physiological artefact) or in EEG where even in gamma band, physiological artefact (e.g., from muscles) dominates.

## Appendix 1 Derivation of the effect of error

This appendix details how to deriveEquation 9, givenEquation 6and the expression for the inverse of the covariance matrix. First note that the beamformer projected source power,$P_{\alpha }$, is given by

$$P_{\alpha }={\text{w}}_{\alpha }^{T}\text{C}{\text{w}}_{\alpha }=\frac{{\text{L}}_{\alpha }^{T}{\text{C}}^{-1}\text{C}{\text{C}}^{-1}{\text{L}}_{\alpha }}{\left({\text{L}}_{\alpha }^{T}{\text{C}}^{-1}{\text{L}}_{\alpha }\right)\left({\text{L}}_{\alpha }^{T}{\text{C}}^{-1}{\text{L}}_{\alpha }\right)}=\frac{1}{{\text{L}}_{\alpha }^{T}{\text{C}}^{-1}{\text{L}}_{\alpha }}$$
[A1]

This allows us to expressEquation 6as

$$\hat{q_{\alpha }}\left(t\right)=q_{\alpha }\left(t\right)+P_{\alpha }{\text{L}}_{\alpha }^{T}{\text{C}}^{-1}\text{e}\left(t\right)$$. [A2]

Now substituting for${\text{C}}^{-1}$inEquation A2, we get

qα^(t)=qα(t)+PαLαT(1ν2(I−fLαLαT‖Lα‖2))e(t)=qα(t)+Pαν2(LαTe(t)−fLαTLαLαTe(t)‖Lα‖2),
[A3]

which, noting that${\text{L}}_{\alpha }^{T}{\text{L}}_{\alpha }={\left\|{\text{L}}_{\alpha }\right\|}^{2}$, simplifies to

qα^(t)=qα(t)+Pαν2(LαTe(t)−fLαTe(t))=qα(t)+Pαν2LαTe(t)(1−f).
[A4]

Now revisitingEquation A1, we note that

Pα−1=LαTC−1Lα=LαT(1ν2(I−fLαLαT‖Lα‖2))Lα=1ν2(LαTLα−fLαTLαLαTLα‖Lα‖2),
[A5]

which simplifies to

$$P_{\alpha }^{-1}=\frac{{\left\|{\text{L}}_{\alpha }\right\|}^{2}}{\nu^{2}}\left(1-f\right)$$. [A6]

Now substituting for$P_{\alpha }$inEquation A4, we get

$$\hat{q_{\alpha }}\left(t\right)=q_{\alpha }\left(t\right)+\frac{\frac{1}{\nu^{2}}{\text{L}}_{\alpha }^{T}\text{e}\left(t\right)\left(1-f\right)}{\frac{{\left\|{\text{L}}_{\alpha }\right\|}^{2}}{\nu^{2}}\left(1-f\right)}=q_{\alpha }\left(t\right)+\frac{{\text{L}}_{\alpha }^{T}\text{e}\left(t\right)}{{\left\|{\text{L}}_{\alpha }\right\|}^{2}}$$. [A7]

If we then lete(t)=νε(t)whereε(t)is a gaussian random process with unit standard deviation, we arrive at the expression,

$$\hat{q_{\alpha }}\left(t\right)=q_{\alpha }\left(t\right)+\frac{\nu }{{\left\|{\text{L}}_{\alpha }\right\|}^{2}}{\text{L}}_{\alpha }^{T}\varepsilon \left(\text{t}\right)$$
[A8]

## Appendix 2 A conventional MEG benchmark

To provide a benchmark for SNR, we again employed the naturalistic viewing task, and undertook similar experiments to those described above (in the same subject) using conventional MEG instrumentation. All data were recorded with a 275-channel axial gradiometer SQUID based system (CTF, Vancouver, Canada) operating in third-order synthetic gradiometer configuration, with a sampling frequency of 600 Hz. The SQUID-MEG system is housed in a MSR (Vacuumschmelze, Hanau, Germany) to reduce the effects of external interference. As with all conventional MEG recordings, the subject was asked to remain still throughout data acquisition. Prior to entering the scanner, three head position indicator (HPI) coils were placed on the subject’s head, and their locations inside the MEG helmet were tracked continuously throughout the recording. Prior to the experiment, a 3D digitiser (Polhemus, Colchester, Vermont, USA) was used to measure the location of the HPI coils relative to the head. The digitised surface was then fitted to the equivalent surface extracted from the anatomical MRI. This enabled co-registration of the MEG sensor geometry to brain anatomy. As with the OPM recordings, the subject underwent this experiment 6 times; the OPM and CTF recordings were interleaved and carried out at approximately the same time each day. The stimulus was identical as that used for OPM (and described above) and was matched for visual angle. However, the projector used to present the visual stimulus was different, likewise the viewing angle and ambient background lighting (these differences were unfortunately unavoidable).

Conventional MEG data were processed using a beamformer technique; data covariance was computed in the 52-65 Hz band, and a dipole model was constructed using a multi-sphere head model. A pseudo-T-statistical image was generated, and time courses of narrow band (52-65 Hz) electrical activity generated in exactly the same way as for the OPM data. SNR values were also computed in the same way.

Results are shown inFigure A1. The line plots show the envelope of gamma-band activity for all six runs overlaid. In the images, the ellipsoid shows the spread of peak locations from the functional images. Note this is slightly larger for the conventional MEG system, though this could be error due to co-registration.

Both the cryogenic and OPM data show a clear increase in gamma during visual stimulation. For the individual runs, the mean SNR of the cryogenic MEG was 2.3 ± 0.5. For the OPM-MEG using all sensors, it was significantly (0.013; Wilcoxon sum rank test) higher at 3.0 ± 0.2. When averaging the envelopes from each run together, the group-level SNR was 4.6 for the cryogenic MEG and 6.8 for the OPMs. These data show that SNR of OPMs compares favourably to SQUIDs—though as reported in our discussion, there are significant caveats relating to experimental set up. Most importantly, it is clear from the linear increase in SNR with total signal that continuing to increase the sensor density of the OPM array will allow it to significantly improve far beyond what can be achieved using cryogenic MEG.

## References

[b1] Almeida , A. N. , Martinez , V. , & Feindel , W. ( 2005 ). The first case of invasive EEG monitoring for the surgical treatment of epilepsy: Historical significance and context . Epilepsia , 46 ( 7 ), 1082 – 1085 . 10.1111/j.1528-1167.2005.66404.x 16026560

[b2] Baillet , S. ( 2017 ). Magnetoencephalography for brain electrophysiology and imaging . Nature Neuroscience , 20 ( 3 ), 327 – 339 . 10.1038/nn.4504 28230841

[b3] Beltrachini , L. , von Ellenrieder , N. , Eichardt , R. , & Haueisen , J. ( 2021 ). Optimal design of on‐scalp electromagnetic sensor arrays for brain source localisation . Human Brain Mapping , 42 ( 15 ), 4869 – 4879 . 10.1002/hbm.25586 34245061 PMC8449117

[b4] Berger , H. ( 1929 ). Uber das elektroenkephalogramm des menschen . Archiv Fur Psychiatrie Und Nervenkrankheiten , 87 ( 1 ), 527 – 570 . 10.1007/BF01797193

[b5] Borna , A. , Carter , T. R. , Colombo , A. P. , Jau , Y.-Y. , McKay , J. , Weisend , M. , Taulu , S. , Stephen , J. M. , & Schwindt , P. D. D. ( 2020 ). Non-invasive functional-brain-imaging with an OPM-based magnetoencephalography system . PLoS One , 15 ( 1 ), e0227684 . 10.1371/journal.pone.0227684 31978102 PMC6980641

[b6] Boto , E. , Bowtell , R. , Krüger , P. , Fromhold , T. M. , Morris , P. G. , Meyer , S. S. , Barnes , G. R. , & Brookes , M. J. ( 2016 ). On the potential of a new generation of magnetometers for MEG: A beamformer simulation study . PLoS One , 11 ( 8 ), e0157655 . 10.1371/journal.pone.0157655 27564416 PMC5001648

[b7] Boto , E. , Meyer , S. S. , Shah , V. , Alem , O. , Knappe , S. , Kruger , P. , Fromhold , T. M. , Lim , M. , Glover , P. M. , Morris , P. G. , Bowtell , R. , Barnes , G. R. , & Brookes , M. J. ( 2017 ). A new generation of magnetoencephalography: Room temperature measurements using optically-pumped magnetometers . NeuroImage , 149 , 404 – 414 . 10.1016/j.neuroimage.2017.01.034 28131890 PMC5562927

[b8] Boto , E. , Seedat , Z. A. , Holmes , N. , Leggett , J. , Hill , R. M. , Roberts , G. , Shah , V. , Fromhold , T. M. , Mullinger , K. J. , Tierney , T. M. , Barnes , G. R. , Bowtell , R. , & Brookes , M. J. ( 2019 ). Wearable neuroimaging: Combining and contrasting magnetoencephalography and electroencephalography . NeuroImage , 201 , 116099 . 10.1016/j.neuroimage.2019.116099 31419612 PMC8235152

[b9] Boto , E. , Shah , V. , Hill , R. M. , Rhodes , N. , Osborne , J. , Doyle , C. , Holmes , N. , Rea , M. , Leggett , J. , Bowtell , R. , & Brookes , M. J. ( 2022 ). Triaxial detection of the neuromagnetic field using optically-pumped magnetometry: Feasibility and application in children . NeuroImage , 252 , 119027 . 10.1016/j.neuroimage.2022.119027 35217205 PMC9135302

[b10] Brookes , M. J. , Boto , E. , Rea , M. , Shah , V. , Osborne , J. , Holmes , N. , Hill , R. M. , Leggett , J. , Rhodes , N. , & Bowtell , R. ( 2021 ). Theoretical advantages of a triaxial optically pumped magnetometer magnetoencephalography system . NeuroImage , 236 , 118025 . 10.1016/j.neuroimage.2021.118025 33838266 PMC8249355

[b11] Brookes , M. J. , Leggett , J. , Rea , M. , Hill , R. M. , Holmes , N. , Boto , E. , & Bowtell , R. ( 2022 ). Magnetoencephalography with optically pumped magnetometers (OPM-MEG): The next generation of functional neuroimaging . Trends in Neurosciences , 45 ( 8 ), 621 – 634 . 10.1016/j.tins.2022.05.008 35779970 PMC10465236

[b12] Brookes , M. J. , Vrba , J. , Robinson , S. E. , Stevenson , C. M. , Peters , A. M. , Barnes , G. R. , Hillebrand , A. , & Morris , P. G. ( 2008 ). Optimising experimental design for MEG beamformer imaging . NeuroImage , 39 ( 4 ), 1788 – 1802 . 10.1016/j.neuroimage.2007.09.050 18155612

[b13] Brunet , N. M. , & Fries , P. ( 2019 ). Human visual cortical gamma reflects natural image structure . NeuroImage , 200 , 635 – 643 . 10.1016/j.neuroimage.2019.06.051 31247299 PMC6703910

[b14] Chen , Y. , & Farivar , R. ( 2020 ). Natural scene representations in the gamma band are prototypical across subjects . NeuroImage , 221 , 117010 . 10.1016/j.neuroimage.2020.117010 32505697

[b15] Claus , S. , Velis , D. , Lopes da Silva , F. H. , Viergever , M. A. , & Kalitzin , S. ( 2012 ). High frequency spectral components after secobarbital: The contribution of muscular origin—A study with MEG/EEG . Epilepsy Research , 100 ( 1–2 ), 132 – 141 . 10.1016/j.eplepsyres.2012.02.002 22476037

[b16] Cohen , D. ( 1968 ). Magnetoencephalography: Evidence of magnetic fields produced by alpha-rhythm currents . Science , 161 ( 3843 ), 784 – 786 . 10.1126/science.161.3843.784 5663803

[b17] Engels , M. M. A. , van der Flier , W. M. , Stam , C. J. , Hillebrand , A. , Scheltens , Ph. , & van Straaten , E. C. W. ( 2017 ). Alzheimer’s disease: The state of the art in resting-state magnetoencephalography . Clinical Neurophysiology , 128 ( 8 ), 1426 – 1437 . 10.1016/j.clinph.2017.05.012 28622527

[b18] Feys , O. , Corvilain , P. , Aeby , A. , Sculier , C. , Holmes , N. , Brookes , M. , Goldman , S. , Wens , V. , & De Tiège , X. ( 2022 ). On-scalp optically pumped magnetometers versus cryogenic magnetoencephalography for diagnostic evaluation of epilepsy in school-aged children . Radiology , 304 ( 2 ), 429 – 434 . 10.1148/radiol.212453 35503013

[b19] Hill , R. M. , Boto , E. , Rea , M. , Holmes , N. , Leggett , J. , Coles , L. A. , Papastavrou , M. , Everton , S. K. , Hunt , B. A. E. , Sims , D. , Osborne , J. , Shah , V. , Bowtell , R. , & Brookes , M. J. ( 2020 ). Multi-channel whole-head OPM-MEG: Helmet design and a comparison with a conventional system . NeuroImage , 219 , 116995 . 10.1016/j.neuroimage.2020.116995 32480036 PMC8274815

[b20] Hillebrand , A. , Singh , K. D. , Holliday , I. E. , Furlong , P. L. , & Barnes , G. R. ( 2005 ). A new approach to neuroimaging with magnetoencephalography . Human Brain Mapping , 25 ( 2 ), 199 – 211 . 10.1002/hbm.20102 15846771 PMC6871673

[b21] Hoogenboom , N. , Schoffelen , J.-M. , Oostenveld , R. , Parkes , L. M. , & Fries , P. ( 2006 ). Localizing human visual gamma-band activity in frequency, time and space . NeuroImage , 29 ( 3 ), 764 – 773 . 10.1016/j.neuroimage.2005.08.043 16216533

[b22] Iivanainen , J. , Stenroos , M. , & Parkkonen , L. ( 2017 ). Measuring MEG closer to the brain: Performance of on-scalp sensor arrays . NeuroImage , 147 , 542 – 553 . 10.1016/j.neuroimage.2016.12.048 28007515 PMC5432137

[b23] Iivanainen , J. , Zetter , R. , & Parkkonen , L. ( 2020 ). Potential of on‐scalp MEG: Robust detection of human visual gamma‐band responses . Human Brain Mapping , 41 ( 1 ), 150 – 161 . 10.1002/hbm.24795 31571310 PMC7267937

[b24] Kayser , C. , Salazar , R. F. , & König , P. ( 2003 ). Responses to natural scenes in Cat V1 . Journal of Neurophysiology , 90 ( 3 ), 1910 – 1920 . 10.1152/jn.00195.2003 12750423

[b25] LONDON CITY TOUR . ( 2022 , August 12). gb Brighton Summer Walk Tour, Bustling Brighton Town Centre 4 K [Video] . YouTube. https://youtu.be/K0SvrAfrSNI

[b26] Muthukumaraswamy , S. D. ( 2013 ). High-frequency brain activity and muscle artifacts in MEG/EEG: A review and recommendations . Frontiers in Human Neuroscience , 7 , 138 . 10.3389/fnhum.2013.00138 23596409 PMC3625857

[b27] Nolte , G. ( 2003 ). The magnetic lead field theorem in the quasi-static approximation and its use for magnetoencephalography forward calculation in realistic volume conductors . Physics in Medicine and Biology , 48 ( 22 ), 3637 . 10.1088/0031-9155/48/22/002 14680264

[b28] Nugent , A. C. , Benitez Andonegui , A. , Holroyd , T. , & Robinson , S. E. ( 2022 ). On-scalp magnetocorticography with optically pumped magnetometers: Simulated performance in resolving simultaneous sources . Neuroimage: Reports , 2 ( 2 ), 100093 . 10.1016/j.ynirp.2022.100093 35692456 PMC9186482

[b29] Oostenveld , R. , Fries , P. , Maris , E. , & Schoffelen , J.-M. ( 2011 ). FieldTrip: Open source software for advanced analysis of MEG, EEG, and invasive electrophysiological data . Computational Intelligence and Neuroscience , 2011 , 156869 . 10.1155/2011/156869 21253357 PMC3021840

[b30] Pfurtscheller , G. , & Lopes da Silva , F. H. ( 1999 ). Event-related EEG/MEG synchronization and desynchronization: Basic principles . Clinical Neurophysiology , 110 ( 11 ), 1842 – 1857 . 10.1016/S1388-2457(99)00141-8 10576479

[b31] Pratt , E. J. , Ledbetter , M. , Jiménez-Martínez , R. , Shapiro , B. , Solon , A. , Iwata , G. Z. , Garber , S. , Gormley , J. , Decker , D. , Delgadillo , D. , Dellis , A. T. , Phillips , J. , Sundar , G. , Leung , J. , Coyne , J. , McKinley , M. , Lopez , G. , Homan , S. , Marsh , L. , … Alford , J. K. ( 2021 ). Kernel Flux: A whole-head 432-magnetometer optically-pumped magnetoencephalography (OP-MEG) system for brain activity imaging during natural human experiences . Optical and Quantum Sensing and Precision Metrology , 11700 , 162 – 179 . 10.1117/12.2581794

[b32] Rampp , S. , Stefan , H. , Wu , X. , Kaltenhäuser , M. , Maess , B. , Schmitt , F. C. , Wolters , C. H. , Hamer , H. , Kasper , B. S. , Schwab , S. , Doerfler , A. , Blümcke , I. , Rössler , K. , & Buchfelder , M. ( 2019 ). Magnetoencephalography for epileptic focus localization in a series of 1000 cases . Brain , 142 ( 10 ), 3059 – 3071 . 10.1093/brain/awz231 31373622

[b33] Rea , M. , Boto , E. , Holmes , N. , Hill , R. , Osborne , J. , Rhodes , N. , Leggett , J. , Rier , L. , Bowtell , R. , Shah , V. , & Brookes , M. J. ( 2022 ). A 90‐channel triaxial magnetoencephalography system using optically pumped magnetometers . Annals of the New York Academy of Sciences , 1517 ( 1 ), 107 – 124 . 10.1111/nyas.14890 36065147 PMC9826099

[b34] Rea , M. , Holmes , N. , Hill , R. M. , Boto , E. , Leggett , J. , Edwards , L. J. , Woolger , D. , Dawson , E. , Shah , V. , Osborne , J. , Bowtell , R. , & Brookes , M. J. ( 2021 ). Precision magnetic field modelling and control for wearable magnetoencephalography . NeuroImage , 241 , 118401 . 10.1016/j.neuroimage.2021.118401 34273527 PMC9248349

[b35] Rhodes , N. , Rea , M. , Boto , E. , Rier , L. , Shah , V. , Hill , R. M. , Osborne , J. , Doyle , C. , Holmes , N. , Coleman , S. C. , Mullinger , K. , Bowtell , R. , & Brookes , M. J. ( 2023 ). Measurement of frontal midline theta oscillations using OPM-MEG . NeuroImage , 271 , 120024 . 10.1016/j.neuroimage.2023.120024 36918138 PMC10465234

[b36] Rier , L. , Michelmann , S. , Ritz , H. , Shah , V. , Hill , R. M. , Osborne , J. , Doyle , C. , Holmes , N. , Bowtell , R. , Brookes , M. J. , Norman , K. A. , Hasson , U. , Cohen , J. D. , & Boto , E. ( 2022 ). Test-retest reliability of the human connectome: An OPM-MEG study . BioRxiv . 10.1101/2022.12.21.521184 PMC12007552 40799699

[b37] Robinson , S. , & Vrba , J. ( 1998 ). Functional neuroimaging by synthetic aperture magnetometry . In T. Yoshimoto (Ed.), Recent Advances in Biomagnetism: Proceedings of the 11th International Conference on Biomagnetism , (pp. 302 – 305 ). Held at the Sendai International Conference Center, Sendai, Japan. https://books.google.co.uk/books?id=O5R_PgAACAAJ

[b38] Sadaghiani , S. , Brookes , M. J. , & Baillet , S. ( 2022 ). Connectomics of human electrophysiology . NeuroImage , 247 , 118788 . 10.1016/j.neuroimage.2021.118788 34906715 PMC8943906

[b39] Schofield , H. , Boto , E. , Shah , V. , Hill , R. M. , Osborne , J. , Rea , M. , Doyle , C. , Holmes , N. , Bowtell , R. , Woolger , D. , & Brookes , M. J. ( 2023 ). Quantum enabled functional neuroimaging: The why and how of magnetoencephalography using optically pumped magnetometers . Contemporary Physics , 63 ( 3 ), 161 – 179 . 10.1080/00107514.2023.2182950 PMC10923587 38463461

[b40] Sekihara , K. , & Nagarajan , S. S. ( 2008 ). Adaptive Spatial Filters for Electromagnetic Brain Imaging . Springer Berlin, Heidelberg . 10.1007/978-3-540-79370-0

[b41] Sekihara , K. , Nagarajan , S. S. , Poeppel , D. , & Marantz , A. ( 2004 ). Asymptotic SNR of scalar and vector minimum-variance beamformers for neuromagnetic source reconstruction . IEEE Transactions on Biomedical Engineering , 51 ( 10 ), 1726 – 1734 . 10.1109/TBME.2004.827926 15490820 PMC4041989

[b42] Shah , V. , Doyle , C. , & Osborne , J. ( 2020 ). Zero field parametric resonance magnetometer with triaxial sensitivity (Patent US10775450B1). https://patents.google.com/patent/US10775450B1/en?oq=US16%2f833%2c576

[b43] Takeda , Y. , Gomi , T. , Umebayashi , R. , Tomita , S. , Suzuki , K. , Hiroe , N. , Saikawa , J. , Munaka , T. , & Yamashita , O. ( 2023 ). Sensor array design of optically pumped magnetometers for accurately estimating source currents . NeuroImage , 277 , 120257 . 10.1016/j.neuroimage.2023.120257 37392806

[b44] Taulu , S. , Simola , J. , & Kajola , M. ( 2005 ). Applications of the signal space separation method . IEEE Transactions on Signal Processing , 53 ( 9 ), 3359 – 3372 . 10.1109/TSP.2005.853302

[b45] Tierney , T. M. , Alexander , N. , Mellor , S. , Holmes , N. , Seymour , R. , O’Neill , G. C. , Maguire , E. A. , & Barnes , G. R. ( 2021 ). Modelling optically pumped magnetometer interference in MEG as a spatially homogeneous magnetic field . NeuroImage , 244 , 118484 . 10.1016/j.neuroimage.2021.118484 34418526

[b46] Tierney , T. M. , Holmes , N. , Mellor , S. , López , J. D. , Roberts , G. , Hill , R. M. , Boto , E. , Leggett , J. , Shah , V. , Brookes , M. J. , Bowtell , R. , & Barnes , G. R. ( 2019 ). Optically pumped magnetometers: From quantum origins to multi-channel magnetoencephalography . NeuroImage , 199 , 598 – 608 . 10.1016/j.neuroimage.2019.05.063 31141737 PMC6988110

[b47] Tierney , T. M. , Mellor , S. , O’Neill , G. C. , Timms , R. C. , & Barnes , G. R. ( 2022 ). Spherical harmonic based noise rejection and neuronal sampling with multi-axis OPMs . NeuroImage , 258 , 119338 . 10.1016/j.neuroimage.2022.119338 35636738 PMC10509822

[b48] Tzourio-Mazoyer , N. , Landeau , B. , Papathanassiou , D. , Crivello , F. , Etard , O. , Delcroix , N. , Mazoyer , B. , & Joliot , M. ( 2002 ). Automated anatomical labeling of activations in SPM using a macroscopic anatomical parcellation of the MNI MRI single-subject brain . NeuroImage , 15 ( 1 ), 273 – 289 . 10.1006/nimg.2001.0978 11771995

[b49] Vrba , J. , Robinson , S. , & McCubbib , J. ( 2004 ). How many channels are needed for MEG ? Neurology & Clinical Neurophysiology, 2004 , 99 . https://pubmed.ncbi.nlm.nih.gov/16012656/ 16012656

[b50] Whitham , E. M. , Lewis , T. , Pope , K. J. , Fitzgibbon , S. P. , Clark , C. R. , Loveless , S. , DeLosAngeles , D. , Wallace , A. K. , Broberg , M. , & Willoughby , J. O. ( 2008 ). Thinking activates EMG in scalp electrical recordings . Clinical Neurophysiology , 119 ( 5 ), 1166 – 1175 . 10.1016/j.clinph.2008.01.024 18329954

[b51] Whitham , E. M. , Pope , K. J. , Fitzgibbon , S. P. , Lewis , T. , Clark , C. R. , Loveless , S. , Broberg , M. , Wallace , A. , DeLosAngeles , D. , Lillie , P. , Hardy , A. , Fronsko , R. , Pulbrook , A. , & Willoughby , J. O. ( 2007 ). Scalp electrical recording during paralysis: Quantitative evidence that EEG frequencies above 20 Hz are contaminated by EMG . Clinical Neurophysiology , 118 ( 8 ), 1877 – 1888 . 10.1016/j.clinph.2007.04.027 17574912

[b52] Xia , H. , Ben-Amar Baranga , A. , Hoffman , D. , & Romalis , M. V. ( 2006 ). Magnetoencephalography with an atomic magnetometer . Applied Physics Letters , 89 ( 21 ), 211104 . 10.1063/1.2392722

