# Supplementary material for: Optimising the sensitivity of optically-pumped magnetometer magnetoencephalography to gamma band electrophysiological activity
Source: Imaging Neurosci (Camb). 2024 Mar 19;2:imag-2-00112. doi: 10.1162/imag_a_00112 (PMC12247564; doi:10.1162/imag_a_00112)
Supplement: Supplementary Material [file imag_a_00112-supp.pdf]

Optimising the Sensitivity of Optically-Pumped Magnetometer Magnetoencephalography to Gamma Band Electrophysiological Activity – Supplementary Material

Ryan Hill, Holly Schofield, Elena Boto, Lukas Rier, James Osborne, Cody Doyle, Frank Worcester, Tyler Hayward, Niall Holmes, Richard Bowtell, Vishal Shah, and Matthew Brookes.

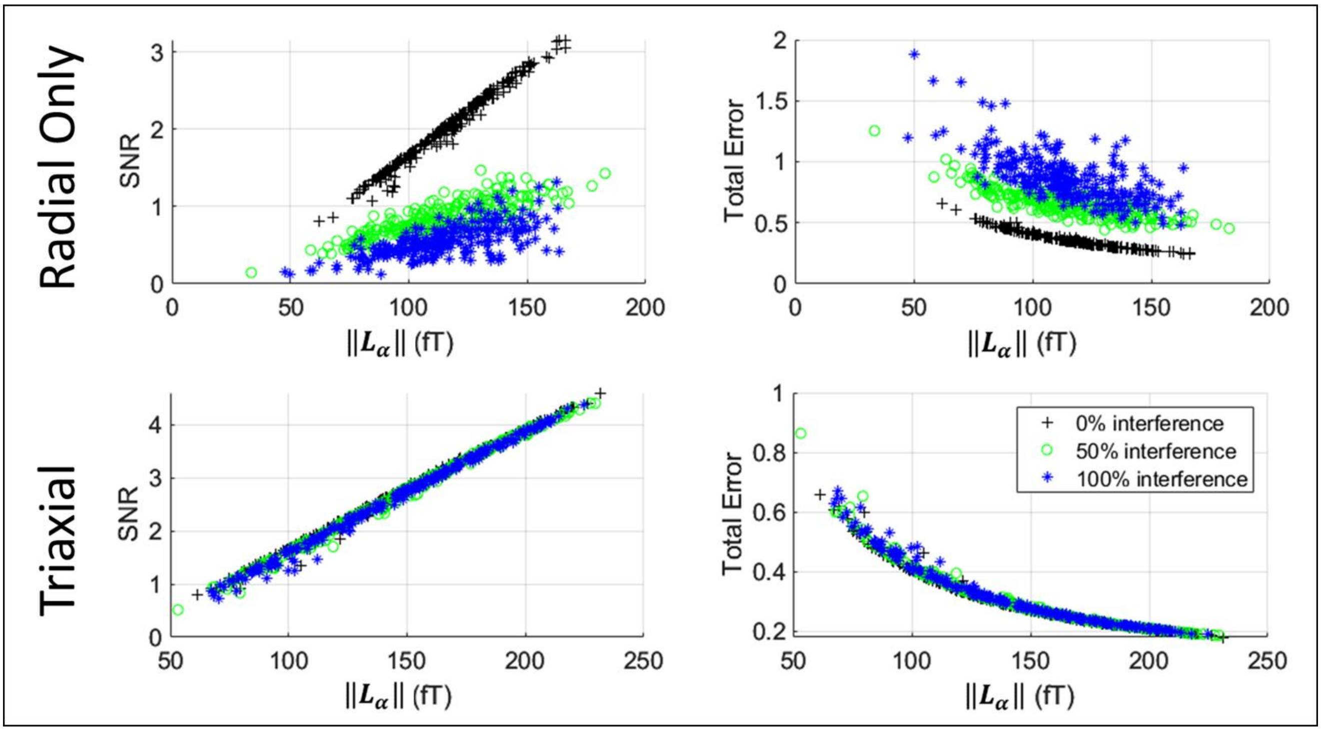

Figure S1: Radial vs Triaxial Simulation Results. *The bottom row is equivalent to the middle panel in Figure 4. The top row shows the same simulation, using radial-only OPMs. Note first that the values for* $||\boldsymbol{L_\alpha}||$ *and SNR are lower for radial sensors – this is simply a consequence of there being fewer channels. However, note also how, with a triaxial array, the presence of external interference has little effect on beamformer reconstructed SNR (the blue, green, and black data points overlap). However, in a radial-only array, the addition of external interference causes a dramatic drop in SNR.*

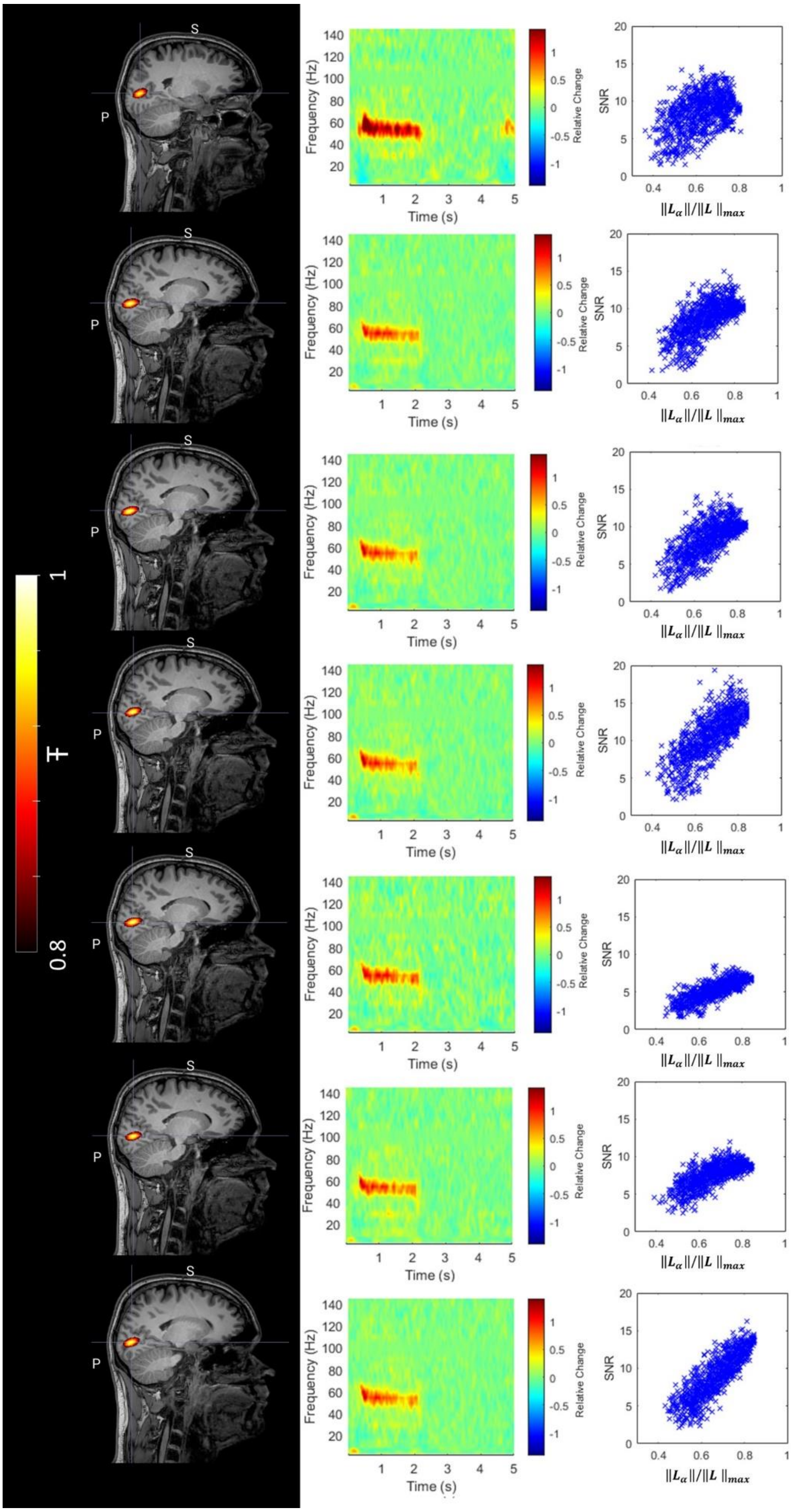

Figure S2: Results from the concentric circles task. *Equivalent to Figure 6 but for the concentric circles task. No equivalent cryogenic data was taken for this task.*
